# Exploring E-cadherin-peptidomimetics interaction using NMR and computational studies

**DOI:** 10.1371/journal.pcbi.1007041

**Published:** 2019-06-03

**Authors:** Monica Civera, Francesca Vasile, Donatella Potenza, Cinzia Colombo, Sara Parente, Chiara Vettraino, Tommaso Prosdocimi, Emilio Parisini, Laura Belvisi

**Affiliations:** 1 Dipartimento di Chimica, Università degli Studi di Milano, Milan, Italy; 2 Istituto di Scienze e Tecnologie Molecolari (ISTM), Consiglio Nazionale delle Ricerche, Milan, Italy; 3 Dipartimento di Scienza e Alta Tecnologia, Università degli Studi dell'Insubria, Como, Italy; 4 Center for Nano Science and Technology @PoliMi, Istituto Italiano di Tecnologia, Milan, Italy; Fox Chase Cancer Center, UNITED STATES

## Abstract

Cadherins are homophilic cell-cell adhesion molecules whose aberrant expression has often been shown to correlate with different stages of tumor progression. In this work, we investigate the interaction of two peptidomimetic ligands with the extracellular portion of human E-cadherin using a combination of NMR and computational techniques. Both ligands have been previously developed as mimics of the tetrapeptide sequence Asp1-Trp2-Val3-Ile4 of the cadherin adhesion arm, and have been shown to inhibit E-cadherin-mediated adhesion in epithelial ovarian cancer cells with millimolar potency. To sample a set of possible interactions of these ligands with the E-cadherin extracellular portion, STD-NMR experiments in the presence of two slightly different constructs, the wild type E-cadherin-EC1-EC2 fragment and the truncated E-cadherin-(Val3)-EC1-EC2 fragment, were carried out at three temperatures. Depending on the protein construct, a different binding epitope of the ligand and also a different temperature effect on STD signals were observed, both suggesting an involvement of the Asp1-Trp2 protein sequence among all the possible binding events. To interpret the experimental results at the atomic level and to probe the role of the cadherin adhesion arm in the dynamic interaction with the peptidomimetic ligand, a computational protocol based on docking calculations and molecular dynamics simulations was applied. In agreement with NMR data, the simulations at different temperatures unveil high variability/dynamism in ligand-cadherin binding, thus explaining the differences in ligand binding epitopes. In particular, the modulation of the signals seems to be dependent on the protein flexibility, especially at the level of the adhesive arm, which appears to participate in the interaction with the ligand. Overall, these results will help the design of novel cadherin inhibitors that might prevent the swap dimer formation by targeting both the Trp2 binding pocket and the adhesive arm residues.

## Author summary

Classical cadherins are the main adhesive proteins at the intercellular junctions and play an essential role in tissue morphogenesis and homeostasis. A large number of studies have shown that cadherin aberrant expression and/or dysregulation often correlate with pathological processes, such as tumor development and progression. Notwithstanding the emerging role played by cadherins in a number of solid tumors, the rational design of small inhibitors targeting these proteins is still in its infancy, likely due to the challenges posed by the development of small drug-like molecules that modulate protein-protein interactions and to the structural complexity of the various cadherin dimerization interfaces that constantly form and disappear as the protein moves along its highly dynamic and reversible homo-dimerization trajectory. In this work, we study the interaction of two small molecules with the extracellular portion of human E-cadherin using a combination of spectroscopic and computational techniques. The availability of molecules interfering in the cadherin homophilic interactions could provide a useful tool for the investigation of cadherin function in tumors, and potentially pave the way to the development of novel alternative diagnostic and therapeutic interventions in cadherin-expressing solid tumors.

## Introduction

Classical cadherins constitute a subfamily of calcium-dependent cell–cell adhesion proteins that belong to the large and phylogenetically diverse cadherin superfamily. The various members of the classical cadherin subfamily show a tissue-dependent expression profile as well as a high sequence and structure homology. In the different tissues, they are mostly localized at the adherens junctions, where they promote cell–cell adhesion through the homodimeric engagement of ectodomains protruding from neighbouring cells [1,2]. This process, which involves cadherin clusterization on the cell membrane, results in the formation of tight cell-cell adhesion interfaces. The extracellular portion of classical cadherins features five tandemly arranged immunoglobulin-like domains (EC1-EC5), whose relative orientation is controlled and rigidified through the coordination of calcium ions at the interdomain level. The interaction of their cytoplasmic tail with catenins allows a number of cell signalling and trafficking processes, providing also a physical link between cadherins and the actin cytoskeleton machinery [3]. The dynamic adhesive interface of the extracellular portion of E-cadherin and other classical cadherins has been revealed by several crystal structures, which so far have captured only some of the numerous conformational states of the protein [4–8]. In essence, dimerization has been shown to involve mainly the two most membrane-distal domains, EC1 and EC2. In order to form the so-called ‘strand-swapped dimer’, two E-cadherin molecules mutually exchange their N-terminal sequence (the A*-strand or adhesion arm), anchoring the aromatic side chain of their Trp2 residue into each other’s binding pocket. Interestingly, to reach the strand swap dimer conformation, which is the reversible endpoint of the dimerization trajectory, two monomeric cadherins must first go through an X-dimer conformation, which lowers the energy of the strand exchange process by firmly placing the two adhesion arms in close physical proximity [9–10].

Beside its broad-ranging effects on physiological tissue organization, classical cadherin dysfunction is often correlated with cancer progression and metastasis [11]. Despite Epithelial (E)- to Neural (N-) cadherin switching being considered a molecular hallmark of epithelial to mesenchymal transition of cancer cells, carcinomas and distal metastases often retain E-cadherin expression [12], as observed for instance in late stage tumors in epithelial ovarian cancer (EOC) [13–15]. In this context, targeting the E-cadherin adhesive interface with small molecules could have a therapeutic and diagnostic value. Linear and cyclic peptides based on the His79-Ala80-Val81 conserved sequence of the EC1 domain (not belonging to the swap dimer interface) have been previously developed to inhibit N-cadherin mediated processes. The most studied cyclic peptide is ADH-1 (Ac-CHAVC-NH_2_), which has been shown to be an anti-angiogenic agent able to cause cell apoptosis [16]. Peptidomimetics of ADH-1 have also been identified [17].

**Fig 1:**
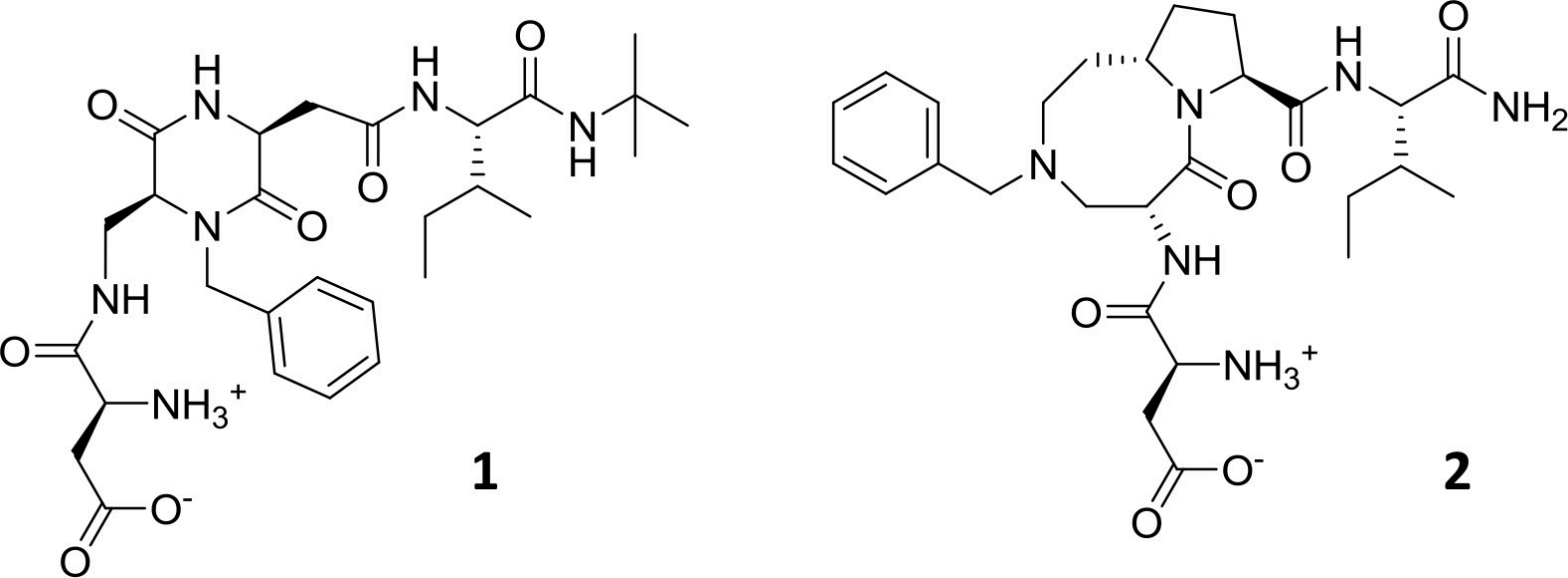
Structures of peptidomimetic ligands 1 and 2.

Recently [18], we reported the first library of small peptidomimetic molecules targeting the strand-swapped dimer interfaces of E- and N-cadherin. These compounds are mimics of the tetrapeptide sequence Asp1-Trp2-Val3-Ile4 (DWVI) of the adhesion arm, modified by replacing the central dipeptide unit (WV) with various scaffolds, all of them bearing a benzyl group that is intended to mimic the indole moiety of Trp2. The most promising compounds identified by a docking protocol were synthesized and were shown to inhibit cadherin homophilic adhesion in EOC cells at low millimolar concentrations. Of these, compound **1** (a.k.a. FR159) (Fig 1), was co-crystallized with a mutant of the E-cadherin EC1-EC2 fragment lacking the first two residues of the adhesion arm (Asp1 and Trp2), while attempts to co-crystallize both compounds **1** and **2** with intact E-cadherin-EC1-EC2 did not yield results [19]. In the X-ray complex structure (PDB code: 4ZTE), compound **1** binds to a dimeric conformation of E-cadherin, the so-called ‘X-dimer’, which is a key intermediate along the E-cadherin dimerization trajectory leading from the monomeric to the strand swap dimer conformation. Unexpectedly, the ligand was found to occupy a hydrophobic pocket that is formed at the X-dimer interface and not in the Trp2 cavity for which it had been designed. This novel hydrophobic pocket formed by the side chains of residues Ile4, Pro5, Ile7, and Val22 from both cadherin molecules does not overlap with the swap dimer interface. However, in the course of the complex cadherin dimerization mechanism the protein undergoes large conformational changes (such as for instance the opening of the adhesion arm that leads to strand swap dimer formation) and goes through different intermediate steps. Hence, it is conceivable that during the process the ligand may bind transiently and via variable moieties also to different surface areas of the protein.

Here, to investigate the extent by which variable ligand-cadherin interactions may form over time in solution and to provide a possible dynamic picture of the binding event, we have applied a combination of NMR (Nuclear Magnetic Resonance) and computational techniques to the complexes of two peptidomimetic inhibitors from our library (Fig 1) and human E-cadherin-EC1-EC2.

We used ligand-based NMR techniques (Saturation Transfer Difference, STD, and transferred NOE, tr-NOESY) [20,21] to assess binding occurrence as well as to identify the binding epitope of the ligands and to estimate the dissociation constant of the complexes. The experiments were performed using wild type (wt) human E-cadherin-EC1-EC2 at three different temperatures (283, 290 and 298 K). Furthermore, we analysed both ligands in the presence of the EC1-EC2 mutant that was used for the X-ray study and that lacks the first two N-terminal residues (E-cadherin-(Val3)-EC1-EC2).

Then, in order to rationalize the atomic details of peptidomimetic-cadherin interaction, the NMR data obtained in the presence of wt E-cadherin-EC1-EC2 were analyzed computationally. Indeed, based on NMR data, docking calculations into the EC1 domain of E-cadherin were carried out first. Then, to take into account the temperature factor and introduce protein flexibility, Molecular Dynamics (MD) simulations were also performed. Two temperatures, 300 K and 320 K, were used starting from the different docking poses of **1** and **2** into the E-cadherin model. Of note, ligands binding seems to be highly dependent on the protein flexibility, particularly of the adhesive arm. Overall, the dynamic data described herein will help the design of novel cadherin inhibitors that may bind more efficiently and more selectively into the Trp2 binding pocket.

## Results

### NMR results

STD-NMR is a consolidated technique [20,21] based on Overhauser effect that is used to study the interactions between small ligands and macromolecules [22–24]. The method relies on the selective irradiation of the protein, which allows magnetization to be transferred to the bound ligand (which is in great excess in solution compared to the protein concentration). The saturated ligand is displaced in solution due to the binding equilibrium and the observation of the ligand signals in the NMR spectrum provides an indication of the interaction. Those ligand protons that are nearest to the protein are more likely to become highly saturated, and therefore show the strongest signal in the mono-dimensional STD spectrum. Owing to the efficiency of the saturation process, the modulation of the ligand signal intensity is used as an epitope-mapping method to describe the target-ligand interactions. In fact, the intensity of the STD signal (expressed as absolute STD percentage) reflects the proximity of the ligand to the protein surface. Therefore, the group epitope mapping obtained provides information about the nature of the chemical moieties of the ligand that are crucial for molecular recognition in the binding site. We also used the STD amplification factors (STD-AF) to derive the dissociation constant (K_D_) of the ligand-protein complexes [25–27]. Moreover, we performed tr-NOESY experiments in order to determine the preferred bound conformation of the ligands.

In solution, the EC1-EC2 domain fragment can adopt several conformations depending on protein and Ca^2+^ ion concentration. At 1 mM calcium concentration and at less than 40 μM protein concentration, a monomeric species is observed predominantly, while dimeric forms or even oligomers are present at higher protein concentrations in solution (600 μM) [28]. The calcium ions provide a rigidification of the linker region connecting the EC1 and EC2 domains in the monomer, thus making the dimerization surface available.

STD–NMR and tr-NOESY spectra were acquired in 20 mM phosphate buffer at pH 7.4 (with 150 mM NaCl and 1 mM CaCl_2_) and 40 μM EC1-EC2 fragments of E-cadherin. In this condition, a monomeric form is predominant in solution [28]. We performed STD-NMR experiments at 283K, 290K and 298K since lowering the temperature helps to shorten the rotational correlation time of the receptor and to increase the effective magnetization transfer. Interestingly, for both compounds we observed different binding epitopes in relation to temperature variations. Furthermore, we also studied the binding modes of the ligands in the presence of the truncated mutant (E-cadherin-(Val3)-EC1-EC2).

#### NMR interaction studies of compound 1 with wt E-cadherin-EC1-EC2

Compound **1** was obtained by replacing the central Trp2-Val3 unit of the DWVI adhesive motif with a diketopiperazine (DKP) scaffold bearing a benzyl ring (Fig 1). Conformational analysis of compound **1** was performed by NOESY and VT (Variable Temperature)-NMR experiments and compared to computational results (S1 and S8 Figs). Owing to the high mobility of the side chains and to the DKP cis configuration, compound **1** can assume a limited number of different conformations, including a “closed” conformation that is evidenced by the presence of weak NOEs between HαAsp and NHtBu and between the aromatic protons and the Ile side chain (a detailed description of the conformational analysis is reported in SI). However, tr-NOESY data show no sign of those NOE contacts that are typical of closed conformation peptides, suggesting that in the bound form compound **1** adopts an extended conformation.

**Fig 2:**
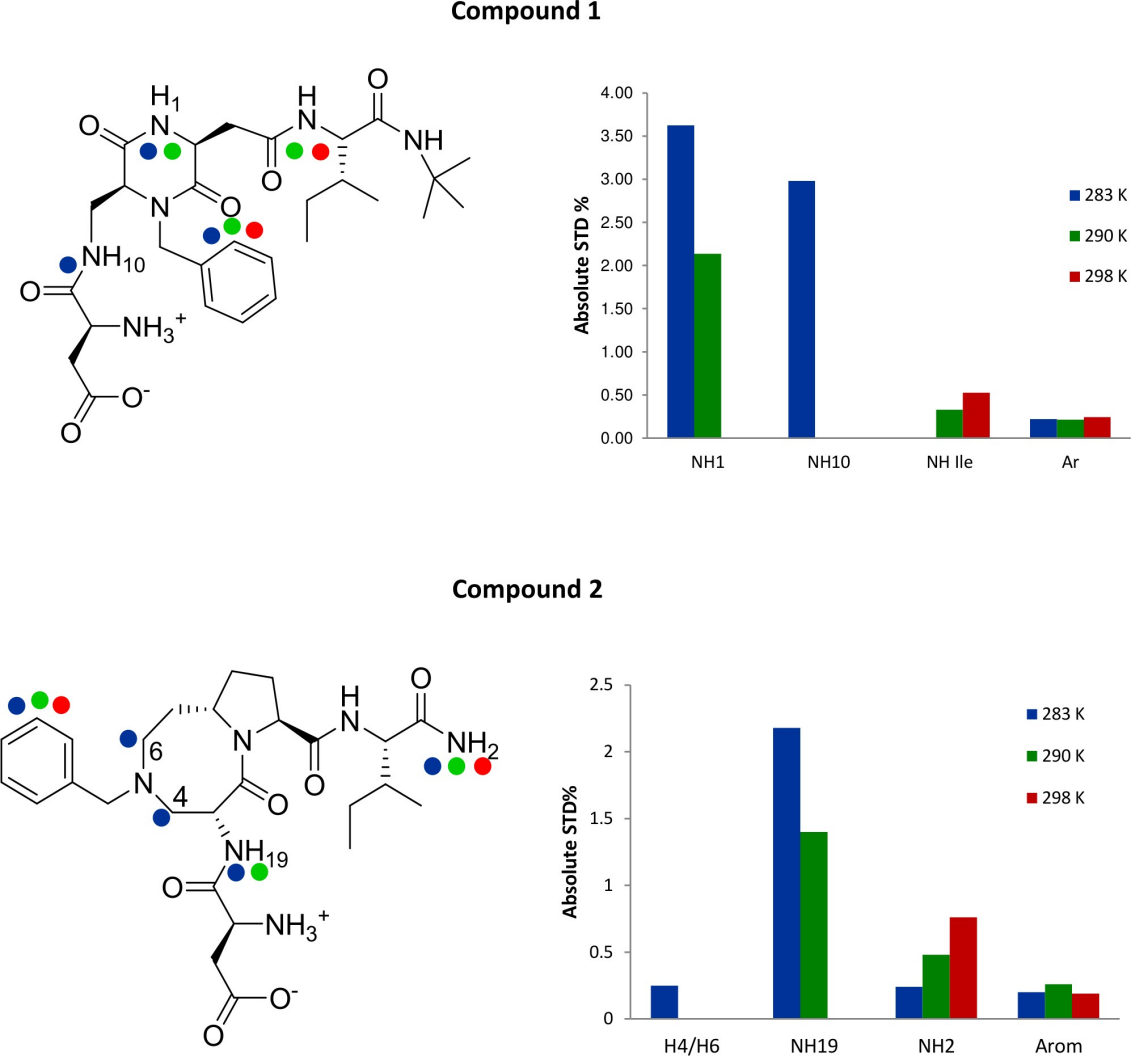
The binding epitope of compounds 1 and 2 in the presence of wt E-cadherin-EC1-EC2. The dots on the structure indicate the ligand binding epitope at different temperatures (left, blu 283K, green 290K and red 298K). The histogram shows the comparison of absolute STD % at different temperatures (right).

The epitope mapping study for compound **1** in the presence of wt E-cadherin-EC1-EC2 was performed by STD at 283, 290 and 298K. Analysis of the STD spectra shows that the aromatic moiety of the ligand interacts at all the temperatures with similar strength, whereas differences in the ligand binding epitope exist for the other moieties at the three different temperatures (Fig 2). The NH_1_ and NH_10_ protons show strong interactions at the lowest temperature, while the NH proton of the Ile backbone seems to interact only at a higher temperature. In particular, at 290K the NH_10_ STD signal disappears, NH_1_ decreases in intensity and the signal of the amidic Ile proton appears (Fig 2 and S2 Fig, S6 Table).

While NHs seem to contribute more effectively than aromatic protons to the binding (Fig 2), it is necessary to consider that in H_2_O the exchangeable protons of the polar side chains in the binding pocket can help transferring the saturation more efficiently, depending also on their exchange rate with bulk water [27].

NMR conformational analysis of compound **1** indicates that epitope variation as a function of the temperature cannot be attributed to specific features of the ligand (see conformational analysis section in SI) but rather to the high flexibility of the protein.

In order to evaluate the interaction strength between E-cadherin and compound **1,** we acquired a series of STD spectra at variable ligand concentrations. The STD amplification factor (STD-AF) can be calculated by multiplying the observed STD by the molar excess of the ligand over the protein [20,21,25–27], so that its values depend on the fraction of bound protein. Therefore, a plot of STD-AF values at increasing ligand concentrations will give rise to the protein-ligand binding isotherm, from which a dissociation constant K_D_ can be derived. Data analysis suggest a K_D_ value in the millimolar range (S3 Fig), which is in agreement with the previously determined inhibition activity observed in adhesion assays performed with E-cadherin-expressing EOC cells [18].

#### NMR interaction studies of compound 1 with E-cadherin-(Val3)-EC1-EC2

**Fig 3:**
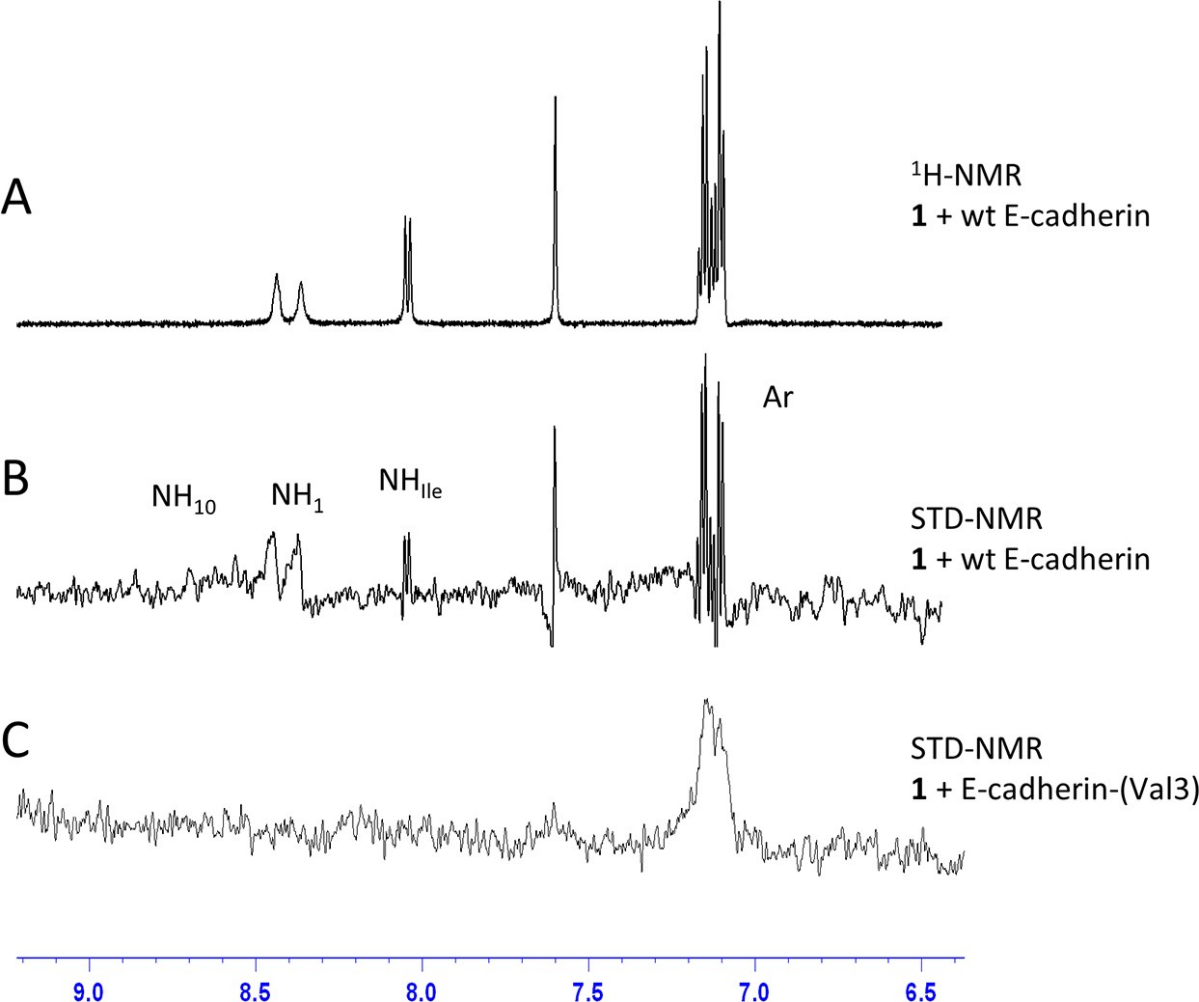
Comparison between the epitopes of compound 1 in the presence of wt and truncated E-cadherin. **A)** ^1^H-NMR at 283K of compound **1**. **B**) and **C**) STD-NMR at 283K of compound **1** in the presence of wt E-cadherin-EC1-EC2 and E-cadherin-(Val3)-EC1-EC2, respectively. The interacting protons are marked.

STD experiments at three different temperatures were also acquired and analysed for compound **1** in the presence of E-cadherin-(Val3)-EC1-EC2. The analysis of STD contacts reveals that only the aromatic protons are able to interact with the protein (Figs 3C and S4). These interactions do not change and are maintained at all temperatures.

The comparison between the STD experiment obtained for compound **1** in the presence of wt (Fig 3B) and truncated E-cadherin (Fig 3C) is shown in Fig 3: the epitopes are very different and in the case of E-cadherin-(Val3)-EC1-EC2 several interactions are lost, suggesting a weaker binding affinity due to the absence of the first two residues of the protein. Additional experiments, which were recorded with a lower amount of D_2_O to reduce H-D exchange for labile protons, confirmed that the amide protons are not involved in binding and in this case the N-terminal amine of the Asp residue of the ligand can be observed (S4 Fig).

#### NMR interaction studies of compound 2 with wt E-cadherin-EC1-EC2

Compound **2** was obtained by replacing the central Trp2-Val3 unit of the DWVI adhesive motif with a conformationally constrained azabicyclic lactam bearing a benzyl ring (Fig 1). Its NOESY spectra acquired in phosphate buffer did not show any significant long range NOE contact typical of a preferred conformation (both at 283K and 298K). In the tr-NOESY spectrum (acquired in the presence of E-cadherin) no long range interactions were detected, as observed for the free-state form, whereas binding evidences of the small ligand to the protein were achieved. Epitope mapping was performed by STD at 283, 290 and 298K (Fig 2, S5 Fig and S7 Table).

The graph of Fig 2 shows the values of absolute STD % of the protons engaged in the interaction with the protein, at the three tested temperatures. The aromatic moiety, which has been introduced to mimic the aromatic side chain of Trp2, interacts always with the protein and the values of absolute STD % are very similar at the different temperatures (about 0.2% STD); this suggests that the aromatic moiety interacts always with the same strength with the protein. On the contrary, as observed for compound **1**, the temperature variation leads to a change in ligand binding epitope for the other moieties: at 283K we observe a strong interaction with NH_19_ (2.078%); also the scaffold (H_4_/H_6_) protons (0.265%), and the C terminal amidic NH_2_ (0.2%) are involved in the binding. At 290 K the strength of the interaction of the scaffold protons decreases (NH_19_ 1.441% while H_4_ and H_6_ are not detectable) and a strong contact for the amidic NH_2_ (0.463%) is observed. At 298K only the aromatic protons and the amidic NH_2_ (0.806%) are observable in the STD spectrum.

Also in this case, the different epitopes are not correlated to a change in the conformation of the ligand (see the conformational analysis section of SI), and could be related to a different behaviour of the protein when the temperature changes.

Also for this compound, the analysis of the STD-AF affords a K_D_ value in the sub-mM range.

#### NMR interaction studies of compound 2 with E-cadherin-(Val3)-EC1-EC2

Compound **2** was analysed at variable temperatures also using E-cadherin-(Val3)-EC1-EC2 as the protein substrate. In this case, the only protons that are involved in the interaction are the aromatic ones. Interestingly, as observed for compound **1**, when this truncated mutant of the protein is used the binding epitope of ligand **2** does not change with the temperature (S6 Fig) and a different ligand binding epitope is observed depending on the cadherin construct. These results confirm that also for compound **2** the N-terminal residues Asp1-Trp2 of the protein are important for the interaction.

### Computational results

The NMR interaction data of peptidomimetics **1** and **2** with the EC1 domain of E-cadherin were further investigated computationally. In the X-ray structure of compound **1** bound to the deleted E-cadherin-(Val3)-EC1-EC2 construct missing the Asp1-Trp2 N-terminal residues, the ligand fits into a novel site at the interface of the X-dimer assembly rather than occupying the Trp2 hydrophobic pocket as initially assumed based on their design strategy. However, according to the NMR results discussed above, the ligands show different binding epitopes depending on the protein construct used, suggesting that the adhesive arm may indeed also be involved in stabilizing the ligand-cadherin binding. To clarify this further, we performed MD simulations starting from the docking poses of **1** and **2** generated into the Trp2 binding pocket of wt E-cadherin model. To take into account temperature modulation, the MD runs were carried out at 300 K and 320 K. For each ligand, a detailed analysis of ligand protonation states and conformational geometries was carried out (see SI) in order to select the most relevant conformations and ionization forms for docking calculations.

#### Docking results of compound 1

**Fig 4:**
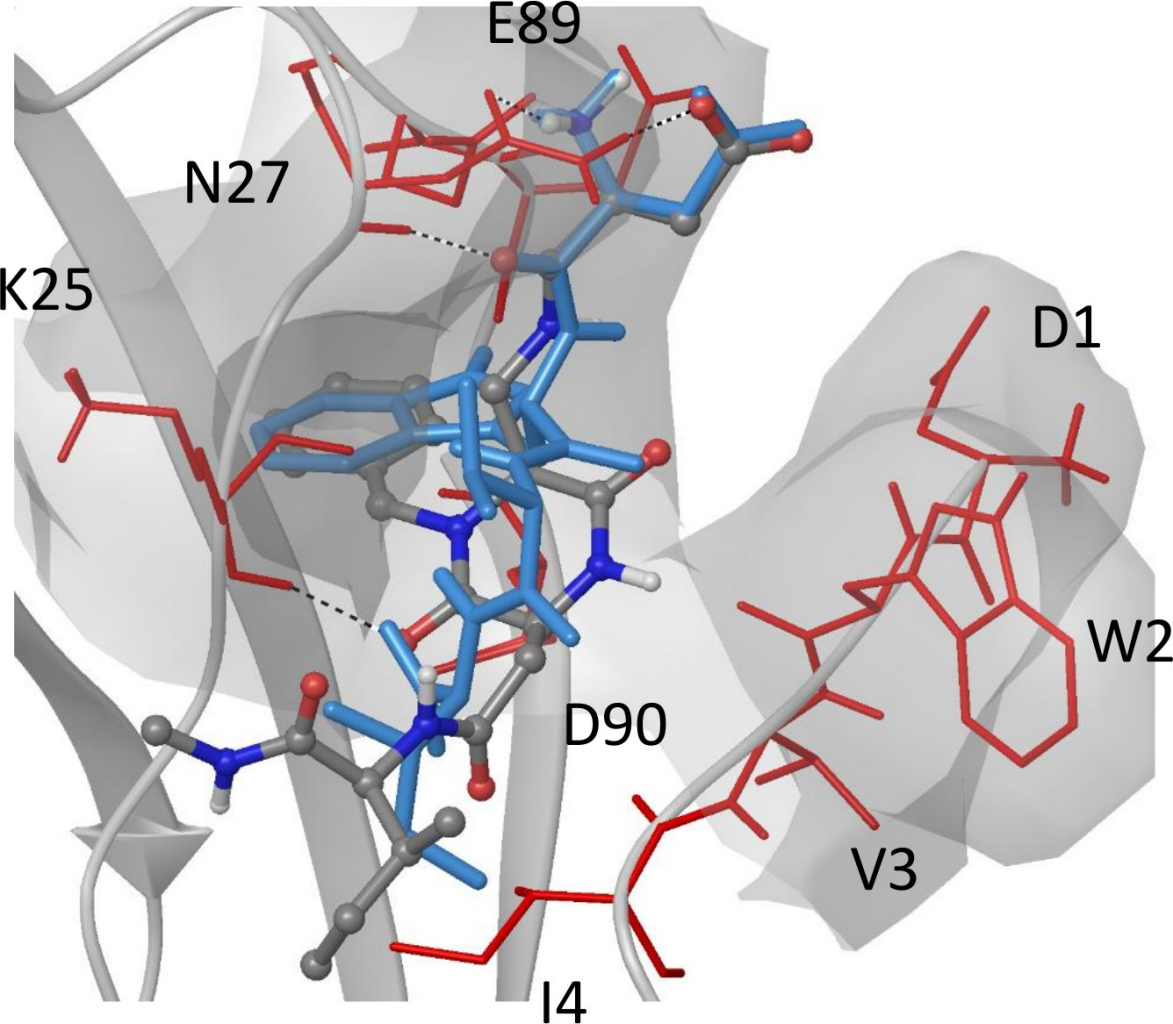
Docking binding mode of the compound 1 into E-cadherin. The ligand (grey carbon atoms) is superimposed to the DWVI sequence (light blue) of the E-cadherin X-ray crystal structure (PDB code: 3Q2V). Residues of the binding pocket interacting with the ligand are labelled.

Docking results of compound **1** into E-cadherin show a good correspondence with the X-ray binding mode of the DWVI native sequence (S10 Fig): the salt bridge of ligand Asp-NH_3_+ with Glu89 (i) is formed and the benzyl ring of the scaffold is fitted into the corresponding hydrophobic pocket (ii). In addition, the C = O group of the DKP scaffold and NH1 form a hydrogen bond with the backbone of Lys25 and Trp2, respectively (Fig 4).

This canonical binding mode, resembling the type **A** binding mode of compound **2** (see discussion below), is maintained in all the docking poses and is in agreement with the binding epitope of the ligand for wt E-cadherin as determined by STD experiments (see SI for details).

Then, MD calculations were carried out at 300 K and 320 K starting from the docking pose (AMBER16 package [29], NVT conditions, AMBER99SB*ildn force field [30] and the explicit water model; see SI and the Experimental section for details). For each temperature, a comparison with docking results and NMR data was performed. In particular, the hydrogen atoms forming the STD epitope of the ligand were analysed in order to determine the neighbouring protein residues (contact analysis) and characterize their interactions.

#### MD simulations at 300K

For the simulations at 300 K, three independent MD runs (200 ns each) were performed using the same input geometry and three different velocities (see SI and the Experimental section for details). The hydrophobic interaction (ii) that is observed in the docking pose is maintained during the whole trajectory (600 ns) and the ligand aromatic ring remains docked into the binding pocket (which is lined by residues Y36, H79, S78, M92 A80, P91, D90, S26, K25, I24, E89). On the contrary, the starting salt bridge with Glu89 (i) is either broken or formed (i.e. it is present in 30% of the sampled structures) and the ligand Asp-NH_3_+ group binds to other charged residues of the binding site, such as the side chains of Asp90 (69%) and Asp1 (38%). In addition, a novel salt bridge is formed between the ligand Asp side chain and the protein Asp1-NH_3_+ N-terminus (38%, see S8 Table).

The ligand and protein RMSD plots show that both molecules move with respect to the starting structure (S11 Fig). The ligand major flexibility is restricted to the DKP arms (see S12 Fig for the ligand cluster analysis), while the protein sampled different conformations of the disordered loop regions (82–90, 25–29 residues of the pocket, and the loop 13–18), and of the adhesive arm (see S12 Fig for the protein cluster analysis). All of them correspond to the most flexible regions of the protein (see RMSF plot in S13 Fig), with the adhesive arm residues showing the highest RMSF fluctuations (especially for residue 1 and 2).

These data are in agreement with the behaviour of the ligand-adhesive arm interactions. In fact, the ligand NH_1_ proton, which forms a hydrogen bond with the Trp2 backbone in the starting structure, establishes new contacts with the residues of the adhesive arm during the simulation, as it becomes engaged by Asp1, Trp2 and Val3 backbone carbonyl groups to form mutually exclusive hydrogen bonds (66% of the sampled structures using a hydrogen-to-acceptor cut-off distance of 3.5 Å).

The contacts analysis of the ligand NH_10_, NH_1,_ and NH_Ile_, protons (Table 1) suggests a different involvement of the residues of the adhesive arm in the interaction with these protons. As discussed in the following, the results of our simulation at 300K well agree with the binding epitope of the ligand at low temperatures. In fact, NH_1_ is the proton that appears to be more involved in the interaction with the protein, especially with the adhesive arm (STD signal detected at 283K and 290K), followed by NH_10_ (detected only at 283 K) and NH_Ile_ (STD signal at 290K and 298 K).

**Table 1:** List of protein residues close to the NH_10_, NH_1_, NH_Ile_ protons of 1 in MD simulations at 300 K and 320 K, ranked according to their most populated contact. Only the percentage value of the most and the least populated contacts is reported.

NH_1_		NH_10_		NH_Ile_	
300 K	320 K	300 K	320 K	300 K	320 K
M92 (88%)	M92 (92%)	D90*(80%)	E89(74%)	W2(36%)	K25* (66%)
V3		M92*	S26*	V3	I24*
W2		W2	N27*	D1*	Q23* (13%)
D1*		P91	D90* (30%)	N27*	
I4 (30%)		D1*		M92*	
		E89		I4 (7%)	
		V3* (16%)			

* new contact formed during MD simulation

#### MD simulation at 320 K

Ligand **1** remains bound to E-cadherin, maintaining both the initial hydrophobic interaction (ii) and the salt bridge with the side chain of Glu89 (i) (83% of the sampled structures, see S8 Table). Rather than engaging with the residues of the adhesive arm, the ligand appears to interact preferentially with the residues of the pocket, especially with Asn27, forming stable hydrogen bonds between Asp-NH_3_+ of the ligand and the Asn27 side chain in 72% of the sampled structures, and between the Asp-C = O moiety of the ligand and the Asn27-NH of the protein (90% populated).

On one hand, the ligands showed a main binding mode (92% of the sampled structures, see S14 Fig) similar to the docking pose but featuring also an intramolecular hydrogen bond between the t-butyl NH amide and the scaffold carbonyl DKP-O1 (28% populated in the MD runs at 300K). On the other hand, the protein sampled different conformations (see S14 Fig for cluster analysis results), mainly differing for the position of the adhesive arm.

By comparing the protein backbone RMSF plots of the simulations at 300 K and 320 K, we observed an increase of the adhesive arm fluctuations with temperature (S13 Fig) while the movements of the other protein loops stay approximately constant (with the exception of residue Asn27, which at 320 K becomes stabilized by ligand interactions).

#### Comparison with STD experiments

As with the MD calculations at 300 K, the aromatic hydrogens of the ligand are in proximity to the residues of the hydrophobic pocket, thus explaining the STD signal of aromatic protons detected at all temperatures. The other protons of the binding epitope are differently engaged by the protein, especially by the residues of the adhesive arm, as a function of the temperature. In the starting structure, NH_10_, NH_1_ and NH_Ile_ make contacts with the protein, but during the simulations at 300 K only NH_10_ and NH_1_ form stable contacts with E-cadherin. For NH_10_ new stable contacts are established with the residues of the binding pocket and the adhesive arm (Asp1 and Val3), and a new hydrogen bond is formed with the backbone of Asp1 (20% populated, see S9 Table). At 320 K, NH_10_ lays close to the pocket residues but loses the starting contact with Trp2 (populated 49% in MD at 300 K) and does not interact with the residues of the adhesive arm. Thus, compared to the MD results at 300K, the interaction of NH_10_ with E-cadherin become weakened, featuring fewer contacts with lower population.

Besides Met92, the NH_1_ contacts at 300 K are mainly due to the hydrogen bonds with the residues of the adhesive arm, while at 320 K they are limited to the pocket and the adhesive arm is not involved in the interaction (population < 4%). At 320 K, also the NH_Ile_ proton loses its interaction with the adhesive arm but the population of the contacts with other residues of the protein increases (from 7–36% to 13–66%).

#### Docking results of compound 2 into E-cadherin

**Fig 5:**
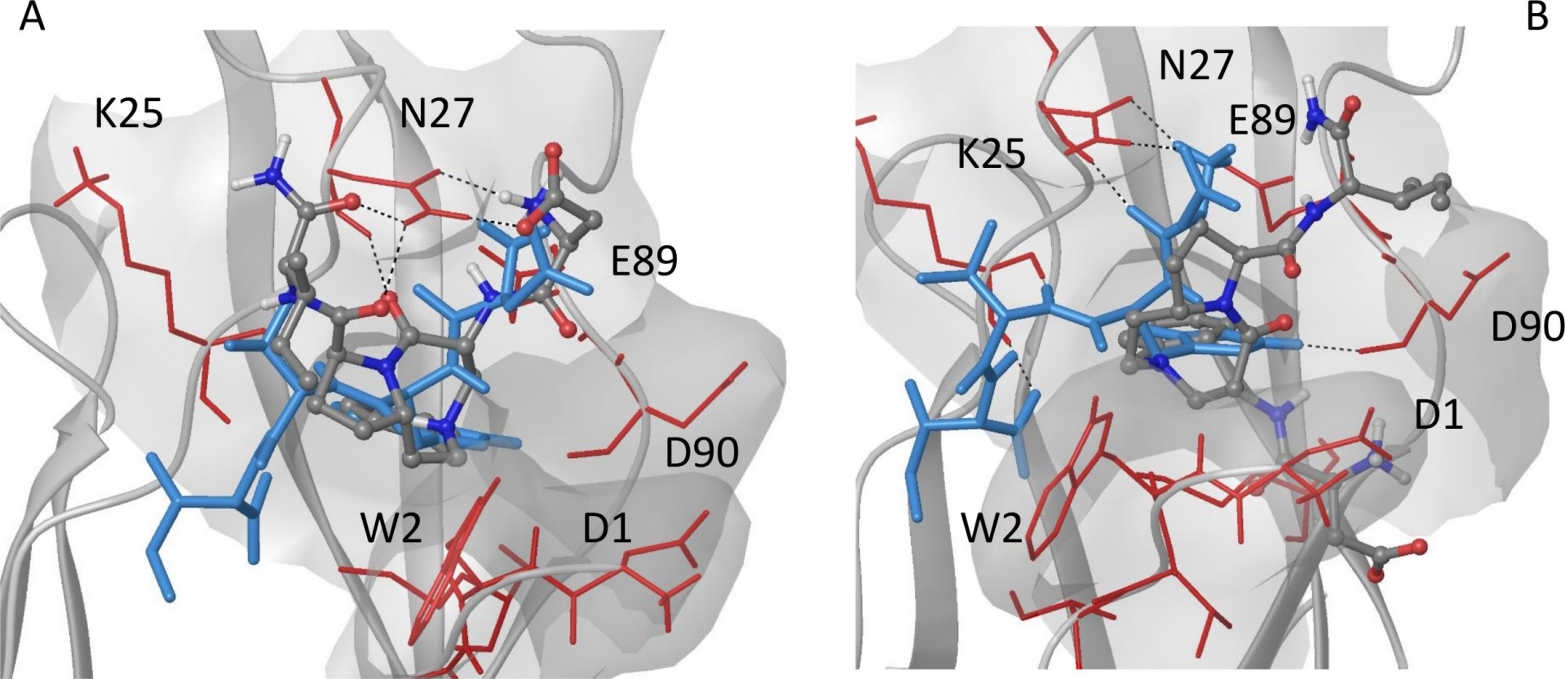
Docking preferred binding modes of compound 2 into E-cadherin. Type **A** is shown for the ligand charged state (left) and type **B** for the ligand neutral state. The compound (grey carbon atoms) is displayed into the E-cadherin X-ray structure (PDB code: 3Q2V) superimposed to the DWVI sequence (light blue). Residues of the binding pocket interacting with the ligand are labelled.

According to Epik results [31], at close to physiological conditions (pH = 7, water solvent) compound **2** is likely to exist as a neutral or positively charged molecule. In fact, these two ionization states of the scaffold cyclic amine, with a calculated pKa of 7.7, contribute equally to the equilibrium. Prior to the docking step, a sampling of the lactam ring conformations was carried out by means of Monte Carlo/Multiple Minimum (MC/MM) calculations [32] (see SI for details) and the global minimum 8,5-ring conformation was used (the same ring geometry was found for all the protonation states). Docking results using the Glide software [33] indicate two possible binding modes, namely **A** and **B** (Fig 5), differently ranked and populated depending on the ligand protonation state.

In the docking poses of type **A**, the salt bridge (i) between the ligand Asp-NH_3_+ and the side chain of E-cadherin Glu89 is formed and the aromatic moiety fits into the hydrophobic pocket of Trp2 (ii), reproducing the X-ray interactions of the native sequence. This canonical binding mode where the ligand Asp and Ile residues adopt a U-shaped conformation around the Asn27 side chain (with both the Asp side chain and the Ile capping group forming hydrogen bonds) is preferred by the charged state (see Fig 5A).

In the docking poses of type **B,** found as top-ranked in the neutral form (Fig 5B), the ligand inserts the benzyl ring into the Trp2 hydrophobic pocket and uses the terminal amide moiety to form a hydrogen bond with Glu89 side chain. The ligand Asp-NH_3_+ is sandwiched between the Asp1 and Asp90 side chains.

Both binding modes and protonation states were selected (4 input complexes) as starting points for MD simulations at 300 K. Only the binding mode **A** of the neutral form appeared to be stable at 300 K and therefore it was selected as the starting point for MD simulations at 320 K.

#### MD simulations at 300 K

The binding of the charged form of compound **2** to E-cadherin is not stable and the ligand aromatic moiety leaves the hydrophobic pocket of the protein at the beginning of the simulations for both types (**A** and **B**) of arrangements. The same behaviour is observed for the neutral state when starting from the binding mode **B**, which corresponds to the best pose, and only the canonical docking pose **A** is maintained. Compared to the starting structure, during the simulation the salt bridge (i) with Glu89 is broken (formed only by 3.5% of the sampled structures) and a new salt bridge between the side chain of the Asp moiety of the ligand and Arg28 of the protein is formed (23%, see S10 Table), whereas the ligand Asp-NH_3_^+^ group forms a new electrostatic interaction with the side chain of Asp1 (54% populated, see S15 Fig). According to the cluster analysis results, this is the most populated, if not the only, binding mode (99%).

In the simulation, the ligand-protein hydrogen bonds of docking pose **A** (highlighted in bold in Table 2) are poorly populated and new hydrogen bonds are sampled, the most relevant involving residues Asp1 (12%) and Trp2 (41%) of the adhesive arm and residue Lys25 of the pocket (54%).

**Table 2:** Populations of ligand-protein hydrogen bonds in MD simulations at 300 K (left) and 320 K (right) for compound 2 (neutral form). The hydrogen bonds of the starting structure (binding mode **A**) are highlighted in bold.

HB (ligand/protein)	Population (%)	
	300 K	320 K
Ile-NH/Lys25-C = O	54	59
Lactam-O3/Trp2-Hε	41	0
Asp-C = O/Trp2-NH	25	0
**Lactam-O4/Asn27-NH**	15	10
Asp-C = O/Asp1-NH_3_+*	12	0
**Asp-OD/Asn27-NH2**	12	5
**NH**_**19**_**/Asn27-OD1**	9	9

According to protein cluster analysis, two main clusters are found (68% and 30% populated, S15 Fig) that differ for the conformation of the loops around the hydrophobic pocket (residues 82–90 and 26–32) and of the adhesive arm residues (residue 1–4) of the protein. In fact, these protein regions show the major fluctuations in the RMSF plot with respect to the starting structure (S13 Fig).

According to the contact analysis of the NH_19_ and NH_2_ protons of the ligand, which show a variation of the STD signals as a function of the temperature, MD simulations at 300 K suggest a greater involvement of NH_19_ in the interaction with E-cadherin compared to NH_2_ protons (Table 3). This behaviour well agrees with the STD spectra at 283 K and 290 K, which are characterized by a higher intensity of the NH_19_ signal relative to NH_2_.

**Table 3:** List of protein residues close to the NH_19_ and NH_2_ protons of 2 in MD simulations at 300 K and 320 K. Only the percentage values of the most and the least populated contacts are reported.

NH_19_		NH_2_	
300 K	320 K	300 K	320 K
N27 (83%)	N27 (88%)	Q23* (20%)	K25* (35%)
W2*	S26	W2*	Q23*
S26	W2*	K25*	I24*
E89	R28	I24*	S26* (16%)
R28	E89 (16%)	N27 (7%)	
D1* (12%)			

* new contact formed during MD simulation

#### MD simulation at 320 K

Docking pose **A** is also stable during the simulation at 320 K, with the aromatic ring of the ligand remaining inside the hydrophobic pocket of the protein. The initial salt bridge (i) with the side chain of Glu89 is lost (< 2% populated) and no other salt bridges involving the ligand N-terminal group are formed. As observed in the MD run at 300 K, the side chain of the Asp moiety of the ligand moves toward the Arg28 side chain to establish a new salt bridge (72% populated, S10 Table). Apart from this movement, the overall orientation of the ligand within the binding site is similar to the input conformation and the ligand cluster results converge to the same single structure (99% populated) resulting from the MD simulations at 300 K. Protein cluster analysis identifies two main clusters (81% and 19% populated), differing for the conformation of the loop region of the protein around the binding site (residues 25 to 33 and 82 to 90) and of the protein adhesion arm (residues 1 to 4), as they coincide with the E-cadherin-EC1-EC2 most flexible regions. Both the RMSD of the protein backbone and the RMSF plots, calculated with respect to the X-ray structure, match well with the 300 K results, indicating a comparable flexibility of the protein (S13 and S16 Figs).

The ligand-protein hydrogen bond analysis highlights that only the interactions with the pocket residues Lys25 and Asn27 are similar with the MD simulations data at 300 K, while the hydrogen bonds with the residues Asp1 and Trp2 of the adhesion arm, which are rather populated at 300 K, are not formed at 320 K.

#### Comparison with STD experiments

The ligand maintains its aromatic ring docked into the hydrophobic pocket at both simulation temperatures, in line with the presence of the corresponding STD signal in all the spectra. The NH_19_ proton, which is involved in a hydrogen bond with the side chain of Asn27 in the docking pose (Table 2), poorly sampled this interaction in both systems (9% of the sampled structures, Table 2). However, during the simulations, this amide proton still maintains the native contacts with the residues of the pocket, especially with Asn27 (>80% populated), and form a new stable contact with Trp2 (70% populated). Compared to MD results at 300 K, the contact with Asp1 is not sampled at 320 K.

The C-terminal NH_2_ hydrogens are less in contact with the protein and the initial contact with Asn27 is not maintained in either system (7% populated at 300 K and not present at 320 K). Novel interactions are formed and, compared to MD results at the lower temperature, an increase of NH_2_ interactions with the residues of the pocket is noted at 320 K (percentage range 16–35 vs. 7–23 at 300 K). In addition, at 320 K the Trp2 residue is excluded from the contacts map.

## Discussion

In this study, we evaluated the binding properties of two peptidomimetic ligands to the extracellular portion of E-cadherin using NMR spectroscopy combined to molecular docking and molecular dynamics calculations. Both ligands have been previously shown to act as mM inhibitors of E-cadherin-mediated cell adhesion [18] and to the best of our knowledge, they are the first peptidomimetics developed from the N-terminal DWVI sequence of the E-cadherin EC1 domain. In these compounds, the central dipeptide unit (Trp2-Val3) of the tetrapeptide motif have been replaced by two different scaffolds bearing an aromatic group that, in our docking model, inserts into the hydrophobic cavity of Trp2, thus preventing swap dimer formation. In accordance with this hypothesis, a reconstruction of the free energy profile of the conformational transition of the E-cadherin monomer from its closed inactive state (with the Trp2 indole moiety intramolecularly docked) to its open form (indole moiety solvent exposed), has shown that in solution the monomer could significantly populate both the open and closed states, which are almost iso-energetic [34]. However, the recent crystallographic structure of **1** in complex with a deleted form of E-cadherin EC1-EC2 domain (lacking the N-terminal Asp1-Trp2 residues) showed also a novel possible mechanism of action based on a different adhesive interface [19]. Indeed, in the X-ray structure the peptidomimetic compound binds to the X-dimer conformation of the protein, a crucial kinetic intermediate of the cadherin dimerization pathway, by inserting into a novel hydrophobic cavity that is formed at the interface of the two interacting cadherins.

Clearly, owing to the complexity of the E-cadherin homo-dimerization process and to the dynamic behaviour of the target itself, which undergoes major conformational changes as part of its substrate recognition mechanism, further investigations of ligand-cadherin binding are needed. In this work, to sample a set of possible interactions of compound **1** and **2** with E-cadherin, we carried out STD-NMR experiments in the presence of two slightly different E-cadherin species, the wt-E-cadherin-EC1-EC2 and the E-cadherin-(Val3)-EC1-EC2 fragments. Depending on the protein constructs, a different binding epitope of the ligand and also a different temperature effect on STD signals were observed, both suggesting an involvement of the Asp1-Trp2 sequence over time and among the set of possible binding events.

Prompted by these considerations, the ligand binding mode proposed by the docking model that places the compound in the Trp2 cavity making interactions with the adhesive arm portion was selected for further investigations. MD simulations were performed starting from the corresponding docking poses of **1** and **2** into a wt-E-cadherin EC1 model. To assess the temperature modulation, MD runs were performed at 300 K and 320 K and the results compared to the STD spectra. The stability of the docking poses and also the interactions with the E-cadherin pocket, including the adhesive arm, were evaluated and compared at the different temperatures. In the input structures, both ligands insert the aromatic group into the Trp2 pocket. The aromatic moiety remains docked into the hydrophobic pocket also during MD runs at 300 K and 320 K, supporting the presence of the aromatic hydrogens STD signal (detected at all temperatures). In general, the starting ligand binding mode is quite conserved during MD run while the protein displays major fluctuations. In particular, for compound **1** protein flexibility increases with temperature, especially for the adhesive arm residues. As a consequence, at 320 K the contacts and the hydrogen bonds of the STD amide protons with the adhesive arm are not present and only the percentage of NH_Ile_ contacts with the pocket residues is enhanced compared to 300 K (Table 1). At 300 K, NH_1_ resulted to be more engaged by the adhesive arm residues, followed by NH_10_ and NH_Ile_ and this behavior agrees with the STD spectrum at 283 K. At 320 K the population of the ligand NH_Ile_ contacts with the protein increased while the other amide protons lost their contacts with the adhesive arm (too flexible) and also reduced the interactions with the residues of the binding site. This trend is in good agreement with the STD spectrum at 298 K where the NH_Ile_ signal replaces those of NH_1_ and NH_10_.

During MD simulations of compound **2,** only the binding mode type **A** is stable and the ligand remains bound to the receptor. At 300 K, the interaction of NH_19_ proton with E-cadherin is stronger than NH_2_ and this behaviour is in agreement with the STD spectra at 283 K and 290 K, showing higher intensity of NH_19_ signals compared to NH_2_. At 320 K, we observed a slight decrease of NH_19_ contacts (Asp1 not present) and, despite losing the interaction with Trp2, an increase of NH_2_ contacts population with the pocket residues. This trend is consistent with the intensity variation observed in the corresponding STD spectra.

In conclusion, our MD results on ligand-cadherin binding are in agreement with STD spectra. The simulations at different temperatures could also explain the different ligand binding epitope observed in the presence of wt E-cadherin. In particular, the modulation of the signals seems to be dependent on the protein flexibility, especially the adhesive arm, an active part of the binding site that participate in the interaction with the ligand. Based on these results, the design of novel cadherin inhibitors to target more efficiently and selectively the Trp2 binding pocket, will be focused on the stabilization of ligand interactions towards both the hydrophobic cavity and the adhesive arm residues. Moreover, further NMR studies with labelled E-cadherin constructs (wt vs. deleted forms) will be carried out to map the protein residues involved in the interaction with the inhibitors and clarify their mechanism of action.

## Materials and methods

### Computational studies

#### Docking calculations

Docking calculations were performed using Glide version 7.0 [33] in the SP, Standard Precision, modality. Receptor grids were built on the EC1 domain of E-cadherin using the X-ray structure of the corresponding swap dimer complex (PDB code: 3Q2V) [8] and prepared as previously described. [18] The ligand sampling was set to ‘Flexible’ with the option ‘Penalize non planar conformation’ for amides and no Epik state penalties were added to the docking score. For each compound, 10 poses were saved after a post-minimization of the ligand structure within the binding site. The docking protocol was initially tested for its ability to reproduce the binding mode of the native DWVI sequence in the crystal. The program was successful in reproducing the experimentally determined binding mode of the peptide as it corresponds to the best-scored pose.

#### MD simulations

Docking poses were used as input structures for MD simulations. The systems were prepared using xleap module of AMBER16 [29] with AMBER99SB*ildn force field [30]. A cubic solvate on box of TIP3P waters [35] (with a buffer of 15 Å) was added and the system was then neutralized by adding Na+ ions (two Na+ ions for compound **1** and the system with the neutral form of compound **2**, one Na+ ion for the system with the charged state of compound **2**). All the calculations were performed with AMBER16 software.

#### Ligands preparation

All the ligands were considered as zwitterions and the AMBER99SBildn* force field was used. The nonstandard residue moieties of the ligands, i.e. the DKP scaffold and the t-butyl capping group of compound **1** and the 8,5-lactam ring of compound **2**, were prepared as new units using the R.E.D. software [36] and the antechamber tool, as described in the SI.

#### System equilibration of the compound 1 and 2 complexes

Each system was first minimized by performing 2000 steps of steepest descent algorithm first keeping the complex fixed (with a harmonic potential, force constant k = 10 kcal/mol Å^2^) and then running an unrestrained minimization on the entire system. The systems were then heated at 300K, first performing 100 ps (time step, dt = 0.5 ps) at constant volume restraining the protein positions (k = 10 kcal/mol Å^2^), then 100ps at constant pressure (p = 1 bar, dt = 0.5 ps) with the Berendsen’s algorithm option for pressure control (relaxation time of 2.0 ps) [37]. For temperature control, the Langevin thermostat was used (collision frequency of 1 ps-1) [38–39]. For the electrostatic forces the Particle Mesh Ewald (PME) method was applied and a cut-off of 9 Å was used for the non-bonded interactions. All bonds involving hydrogen atoms were constrained using the SHAKE algorithm [40].

#### MD set up for compound 1

For the simulations at 300 K three independent MD runs were performed in NVT conditions (dt = 2ps, 200 ns) using the pmemd module. For each run velocities were randomly chosen on the basis of a Maxwellian distribution at 300 K, while coordinates were taken from the last structure of the equilibration step. To constraint all bonds involving hydrogens, the SHAKE algorithm was applied and the Langevin thermostat (collision frequency of 1 ps-1) was used. The PME method with a cut-off of 9 Å for the non-bonded interactions was also used. Structures for analysis were saved every 20 ps. A total of 30,000 structures resulted from the concatenation of the three independent runs.

MD simulations at 320 K were carried out in NVT conditions starting from the coordinates of the equilibrated system at 300 K and applying the same protocol of MD runs at 300 K. A total trajectory of 200 ns was analysed for a total of 10,000 structures.

#### MD set up for compound 2

For the simulation at 300 K, four independent MD runs were performed in NVT conditions using the set up described for compound **1.** Structures for analysis were saved every 20 ps for 200 ns of simulation time. For the ligand neutral state two different starting point were used, the docking pose type **A** and **B**. For the charged state, the pose **A** and **B** were also selected. One MD simulation at 320 K was carried out for the neutral ligand starting from pose **A** (NVT, dt = 2 fs, 200 ns) using the coordinates of the equilibrated system at 300 K and the same calculation set up. A total of 10,000 structures were sampled.

*MD analysis*. The cpptraj utility was used. For the ligand-protein contacts a cut-off radius of 5 Å was applied. The native contacts were calculated considering the minimized structure as reference. Among the atoms belonging to the same protein residue, the contact with the highest percentage was reported in Tables 2 and 3. Only the contacts sampled for more than 5% were listed. Cluster analysis was performed on ligand heavy atoms (CA, N, C, O atoms of Asp and Ile residues and all heavy atoms of DKP and t-butyl unit) and protein Cα atoms. The average linkage method was applied with epsilon value of 1.5 Å.

Protein RMSD and RMSF plots were calculated with respect to the reference minimized structure using the backbone atoms C, N, O, Cα. Ligand RMSDs were performed for the same heavy atoms used for cluster analysis. For the ligand-protein hydrogen bond analysis, two hydrogen bond definitions were used: the default cpptraj criterion (3.0 Å for the acceptor-to-donor heavy atoms distance and 135° for the angle) and the distance cut-off between the donor hydrogen and acceptor heavy atom of 3.5 Å. Only hydrogen bonds with populations higher than 5% were reported. A salt bridge distance criterion was applied, with a cut-off distance between the barycentre of negatively (C-) and positively (N+) charged atoms of 4 Å.

#### Cloning, Expression, and Purification of Human E-cadherin-EC1-EC2

Two different DNA fragments encoding for the EC1-EC2 portion of human E-cadherin, one including residues 1–213 and one lacking the first two N-terminal residues (Asp1-Trp2) (i.e. including residues 3–213) were cloned separately into two pET-3a expression vectors (Novagen) using the NdeI and BlpI restriction sites. In both cases, each fragment was fused at its N terminus to a 6His-tag, a spacer peptide (Ser-Ser-Gly-His-Ile), and the enterokinase recognition site (Asp-Asp-Asp-Asp-Lys). In both DNA constructs, the Cys9Ser mutation was also introduced. Overnight protein expression at room temperature in BL21(DE3)pLysS *E*.*coli* cells (Invitrogen) afforded large quantities of soluble protein for both constructs. Cells were lysed by sonication in TBS, pH 7.4, and 1 mM CaCl_2_. The two cell lysates were purified first by Ni-affinity chromatography and then by gel filtration using a Sephacryl 100 HR HiPrep 26/60 size exclusion column (GE Healthcare). Both proteins were dialyzed in TBS buffer + 20 mM CaCl2, digested with enterokinase (New England Biolabs) at 25°C, and purified again by Ni-affinity chromatography to remove all traces of the cleaved 6His-tag and any residual uncleaved protein. The two flow-through fractions were then collected and further purified by size exclusion chromatography with TBS + 1 mM CaCl2.

### NMR studies

#### Ligand analysis

NMR spectroscopy experiments were performed at 283K and 298K on Bruker Avance 400 and 600 MHz spectrometers. All proton and carbon chemical shifts were assigned unambiguously and the assignments are reported in S1–S4 Tables. The NMR experiments were carried out in an aqueous phosphate (20 mM) buffer pH = 7 with 150 mM NaCl and 1 mM CaCl_2_ in order to observe amide protons with 10% D_2_O. Two-dimensional experiments (TOCSY, NOESY, ROESY and HSQC) were carried out on samples at a concentration range of 3–6 mM. NOESY experiments were performed with a mixing time of 200 and 700 ms.

In order to obtain clear spectra, the water signal suppression was achieved by using excitation sculpting sequence from Bruker library (DPFGSE, Double Pulse Field Gradient Spin Echo).

The conformations of the peptidomimetics were analysed with respect to hydrogen bonding of amide protons (VT-NMR spectroscopy) and NOE contacts [41–42].

#### Binding studies

The spectra were acquired with a Bruker Avance 600 MHz instrument at 283, 290 and 298 K, in a 3 mm NMR tube and in the phosphate buffer previously described (200 μL).

The interaction between ligands and isolated proteins was detected using trNOESY, trROESY and STD experiments. In trNOESY and trROESY experiments, water suppression was achieved by use of an excitation sculpting pulse sequence. In STD experiments water suppression was achieved by using the WATERGATE 3-9-19 pulse sequence.

The on-resonance irradiation of the protein was kept at -0.05 ppm. Off-resonance irradiation of the protein was applied at 200 ppm, where no protein signals were visible. Selective presaturation of the protein was achieved by a train of Gauss shaped pulses of 49 ms length each. The total length of the saturation train was 2.94 s.

All the experiments were conducted with the first two domains (EC1-EC2) of wt E–cadherin (Molecular Weight EC1-EC2 construct = 23 KDa) and with the construct lacking the first two residues (E-cadherin-(Val3)-EC1-EC2), with a ligand-protein ratio of 80:1.

Since calcium ions are important to rigidify the EC1-EC2 linker region in the monomeric protein and considering that the use of phosphate buffer in the presence of CaCl_2_ may be critical, due to the limited solubility of CaHPO_4_, we studied the stability of the protein in phosphate buffer. We monitored the chemical shift variation in ^1^H-^15^N-HSQC experiment using the ^15^N-labeled protein for 24 hours and we can confirm that the protein remains in the correct folding.

For the determination of the dissociation constant (K_D_) at 283 K, the concentration of the protein was kept 0.05 mM while the ligand concentration was increased from 2.0 mM to 16.0 mM in order to obtain different ligand-protein ratios (from 40:1 to 320:1).

The K_D_ value can be obtained from the initial growth rate of the STD amplification factor. Since the STD-NMR intensity reflects the concentration of ligand-receptor complex in solution, Mayer and Meyer proposed [19] a conversion rule from the observed experimental intensities (η = I_0_-I_sat_/I_0_) to the STD amplification factors (STD-AF), that is by multiplying the observed STD by the molar excess of ligand over protein (ε).

$$STD‐AF=\varepsilon (I_{0}‐I_{sat})/I_{0}=\varepsilon \eta_{STD}$$

This converts the STD intensity, which depends on the fraction of bound ligand, into a factor (STD-AF) that is function of the fraction of bound protein. Thus, the evolution of the STD-AF along a ligand titration series enables construction of a saturation curve in the form of an association isotherm. These curves can be adjusted by means of the above equation and the value of K_D_ results from the mathematical fit [f(x) = a*x/(k+x)] of the experimental curve.

## Supporting information

S1 Text NMR and computational data of ligands in the free and bound states. (DOCX)

S1 Fig Proton spectra of compound 1 at different temperatures (aligned on aromatic signals). The interaction between the tert-butyl moiety and the α proton of the aspartic side chain is conserved at both the lower and at the higher temperature.
(TIF)

S2 Fig Comparison of STD spectra of compound 1 at different temperatures in presence of E-cadherin. (TIF)

S3 Fig Variation of STD-AF versus the increase of ligand 1 concentration in presence of E-cadherin interacting protons and the intensities observed are reported in the graph. We measured the K_D_ value of compound 1 (S3 Fig) and 2 in the presence of E-cadherin by STD (for each point we performed two STD measurements). We obtained a K_D_ value of more than 20 mM, demonstrating a low affinity of this compound for the protein.
(TIF)

S4 Fig Comparison between the epitopes of compound 1 in the presence of truncated E-cadherin at different temperatures. A) and C) ^1^H-NMR at 283 K and 298 K of compound **1** in the presence of E-cadherin-(Val3)-EC1EC2, respectively. B) and D) STD-NMR at 283 K and 298 K of compound **1** in the presence E-cadherin-(Val3)-EC1EC2, respectively. The observation of the terminal AspNH_3_+ is possible since we acquired experiments in the absence of D_2_O.
(TIF)

S5 Fig Comparison of STD spectra of compound 2 at different temperatures in presence of E-cadherin. (TIF)

S6 Fig Comparison between the epitopes of compound 2 in the presence of truncated E-cadherin at different temperatures. A) and C) ^1^H-NMR at 283 K and 298 K of compound **2** in the presence of E-cadherin-(Val3)-EC1EC2, respectively. B) and D) STD-NMR at 283 K and 298 K of compound **2** in the presence E-cadherin-(Val3)-EC1EC2, respectively.
(TIF)

S7 Fig Protonation states of compound 2. According to Epik [31], the tertiary scaffold amine of compound **2** (predicted pKa 7.7) is likely to exist as neutral and protonated forms, equally populated, at physiological condition (pH = 7 and water solution).
(TIF)

S8 Fig Representative conformations of compound 1. Left: Most populated 12-membered ring hydrogen bond geometry sampled with AMBER* during MC/SD simulation; Center: MC/MM OPLS_2005 global minimum geometry; Right: 10-membered ring hydrogen bond structure.
(TIF)

S9 Fig Docking pose best poses of the neutral form of compound 2 into E-cadherin x-ray structure. Ligand global minimum ring geometry (grey) and the relative minimum geometry (blue) were shown.
(TIF)

S10 Fig 2D representation of the DWVI interactions into x-ray E-cadherin binding site. The E-cadherin interactions of the DWVI sequence in the X-ray structure of the swap dimer are formed by an intermolecular salt bridge between the charged N-terminal amino group of Asp1 and the side chain of Glu89 (i), the anchoring of the aromatic moiety of Trp2 into a hydrophobic pocket (ii) and the hydrogen bond between the indole NHε and the carbonyl group of Asp90 backbone (iii). Protein residues within 4 Å are shown, PDB CODE: 3Q2V.
(TIF)

S11 Fig Ligand heavy atoms root-mean-square deviation (RMSD, upper level) and protein backbone atoms (C, O, N, Cα, H) RMSD (lower level) of compound 1 calculated with respect to the docking pose at 300 K (red) and 320 K (blue).
(TIF)

S12 Fig Representative clusters (populated > 5%) for compound 1 MD simulations at 300 K. Left: ligand clusters on heavy atoms (#1 = 35%, #2 = 21%, #3 = 14%, #4 = 12% and #5 = 6%) overlaid to the starting geometry (red); Right: protein clusters on Cα atoms (#1 = 40%, #2 = 24%, #3 = 14% and #4 = 6%) overlaid to the starting geometry (red). Flexible loop and adhesive arm residues are indicated.
(TIF)

S13 Fig Protein root-mean-square fluctuation (RMSF C, O, N, Cα, H backbone atoms) of compounds 1 (upper panel) and 2 (lower panel) calculated with respect to the x-ray structure at 300 K (red) and 320 K (blue).
(TIF)

S14 Fig Representative clusters (populated > 5%) for compound 1 MD simulations at 320 K. Left: most populated ligand cluster (#1 = 92%) overlaid to the starting geometry (red); Right: protein clusters on Cα atoms (#1 = 20%, #2 = 10%, #3 = 8%, #4 = 7%, #5 = 7% and #6 = 6%,) overlaid to the starting geometry (red).
(TIF)

S15 Fig Representative clusters for compound 2 MD simulations at 300 K. Left: ligand cluster (on heavy atoms, 99% populated) overlaid to the starting geometry (red); Right: protein clusters (on Cα atoms) overlaid to the starting geometry (red). Flexible loop and adhesive arm residues are indicated.
(TIF)

S16 Fig Protein RMSD (C, O, N, Cα, H backbone atoms) of compound 2 calculated with respect to the x-ray structure at 300 K and 320 K. (TIF)

S1 Table Structure, 1H and 13C assignment and NOE contacts of compound 1 at 283 K. (PDF)

S2 Table Structure, 1H and 13C assignment and NOE contacts of compound 1 at 298 K. (PDF)

S3 Table Structure, 1H and 13C assignment and NOE contacts of compound 2 at 298 K. (PDF)

S4 Table Structure, 1H and 13C assignment and NOE contacts of compound 2 at 283 K. (PDF)

S5 Table Δδ/ΔT values for the amidic proton of compound 1. (PDF)

S6 Table Absolute STD % of compound 1 at different temperatures in the presence of E-cadherin. (PDF)

S7 Table Absolute STD % of compound 2 at different temperature in the presence of E-cadherin. (PDF)

S8 Table Populations of ligand-protein salt bridges for compound 1 at 300 K and 320 K. The starting salt bridge is highlighted in bold.
(PDF)

S9 Table Populations of ligand 1 STD amide protons hydrogen bonds with the E-cadherin adhesive arm residues at 300 K. (PDF)

S10 Table Populations of ligand-protein salt bridges for compound 2 at 300 K and 320 K. The starting salt bridge is highlighted in bold.
(PDF)

## References

[1] Takeichi M. Dynamic contacts: rearranging adherens junctions to drive epithelial remodelling. Nat. Rev. Mol. Cell Biol. 2014; 15: 397–410. 10.1038/nrm3802 24824068

[2] Brash J, Harrison OJ, Honig B, Shapiro L. Thinking outside the cell: how cadherins drive adhesion. Trends Cell. Biol., 2012; 22: 299–310. 10.1016/j.tcb.2012.03.004 22555008 PMC3385655

[3] Gul IS, Hulpiau P, Saeys Y, van Roy F. Evolution and diversity of cadherins and catenins. Exp. Cell Res. 2017; 358: 3–9. 10.1016/j.yexcr.2017.03.001 28268172

[4] Parisini E, Higgins JM, Liu JH, Brenner MB, Wang JH, The crystal structure of human E-cadherin domains 1 and 2, and comparison with other cadherins in the context of adhesion mechanism. J Mol Biol. 2007; 373: 401–411. 10.1016/j.jmb.2007.08.011 17850815 PMC2094043

[5] Dalle Vedove A, Lucarelli AP, Nardone V, Matino A, Parisini E. The X-ray structure of human P-cadherin EC1-EC2 in a closed conformation provides insight into the type I cadherin dimerization pathway. Acta Crystallogr F Struct Biol Commun. 2015; 71: 371–380. 10.1107/S2053230X15003878 25849494 PMC4388168

[6] Harrison OJ, Jin X, Hong S, Bahna F, Ahlsen G, Brasch J et al The extracellular architecture of adherens junctions revealed by crystal structures of type I cadherins. Structure. 2011; 19: 244–256. 10.1016/j.str.2010.11.016 21300292 PMC3070544

[7] Harrison OJ, Bahna F, Katsamba PS, Jin X, Brasch J, Vendome J et al Two-step adhesive binding by classical cadherins. Nat Struct Mol Biol. 2010; 17: 348–357. 10.1038/nsmb.1784 20190754 PMC2872554

[8] Sivasankar S, Tuning the kinetics of cadherin adhesion. J Invest Dermatol. 2013; 133: 2318–2323. 10.1038/jid.2013.229 23812234 PMC3773255

[9] Li Y, Altorelli NL; Bahna F, Honig B, Shapiro L, Palmer AG. Mechanism of E-cadherin dimerization probed by NMR relaxation dispersion. Proc. Natl. Acad. Sci. USA. 2013; 110: 16462–16467 10.1073/pnas.1314303110 24067646 PMC3799306

[10] Priest AV, Shafraz O, Sivasankar S. Biophysical basis of cadherin mediated cell-cell adhesion. Exp Cell Res. 2017; 358: 10–13. 10.1016/j.yexcr.2017.03.015 28300566

[11] Berx G, van Roy F. Involvement of members of the cadherin superfamily in cancer. Cold Spring Harb. Perspect. Biol. 2009; a003129 10.1101/cshperspect.a003129 20457567 PMC2882122

[12] Wong SHM, Fang CM, Chuah LH, Leong CO, Ngai SC. E-cadherin: Its dysregulation in carcinogenesis and clinical implications. Crit Rev Oncol Hematol. 2018; 121:11–22. 10.1016/j.critrevonc.2017.11.010 29279096

[13] Xu S, Yang Y, Dong L, Qiu W, Yang L, Wang X et al Construction and characteristics of an E-cadherin-related three-dimensional suspension growth model of ovarian cancer. Sci. Rep. 2014; 4: 5646 10.1038/srep05646 25008268 PMC5381612

[14] Roggiani F, Mezzanzanica D, Rea K, Tomassetti A. Guidance of Signaling Activations by Cadherins and Integrins in Epithelial Ovarian Cancer Cells. Int. J. Mol. Sci. 2016; 17: 1387. 10.3390/ijms17091387 PMC5037667 27563880

[15] De Santis G, Miotti S, Mazzi M, Canevari S, Tomassetti A. E-cadherin directly contributes to PI3K/AKT activation by engaging the PI3K-p85 regulatory subunit to adherens junctions of ovarian carcinoma cells. Oncogene. 2009; 28: 1205–1217. 10.1038/onc.2008.470 19151754

[16] Blaschuk OW, N-cadherin antagonists as oncology therapeutics. Philos Trans R Soc Lond B Biol Sci. 2015; 370: 20140039 10.1098/rstb.2014.0039 25533096 PMC4275908

[17] Gour BJ, Blaschuk OW, Ali A, Ni F, Chen Z, Michaud S et al. 2007. United States Patent Number 7, 268, 115.

[18] Doro F, Colombo C, Alberti C, Arosio D, Belvisi L, Casagrande C et al Computational design of novel peptidomimetic inhibitors of cadherin homophilic interactions. Org. Biomol. Chem. 2015; 13: 2570–2573. 10.1039/c4ob02538e 25614037

[19] Nardone V, Lucarelli AP, Dalle Vedove A, Fanelli R, Tomassetti A, Belvisi L et al Crystal Structure of Human E-Cadherin-EC1-EC2 in Complex with a Peptidomimetic Competitive Inhibitor of Cadherin Homophilic Interaction. J.Med.Chem. 2016; 59: 5089–5094. 10.1021/acs.jmedchem.5b01487 27120112

[20] Mayer M, Meyer B. Group epitope mapping by saturation transfer difference NMR to identify segments of a ligand in direct contact with a protein receptor. J. Am. Chem. Soc. 2001; 123: 6108–6117. 11414845 10.1021/ja0100120

[21] Meyer B, Peters T. NMR spectroscopy techniques for screening and identifying ligand binding to protein receptors. Angew. Chem. In. Ed. 2003; 42: 864–890. 10.1002/anie.200390233 12596167

[22] Potenza D, Belvisi L, Vasile F, Moroni E, Cossu F, Seneci P. A NMR and computational study of Smac mimics targeting both the BIR2 and BIR3 domains in XIAP protein. Org. Biomol. Chem. 2012; 10: 3278–3287. 10.1039/c2ob06979b 22407164

[23] Vasile F, Rossi D, Collina S, Potenza D. Diffusion-Ordered Spectroscopy and Saturation Transfer Difference NMR Spectroscopy Studies of Selective Interactions between ELAV Protein Fragments and a mRNA Target. Eur. J. Org. Chem. 2014; 29: 6399–6404.

[24] Vasile F, Gubinelli F, Panigada M, Soprana E, Siccardi A, Potenza D. NMR interaction studies of Neu5Ac-α-(2,6)-Gal-β-(1–4)-GlcNAc with influenza-virus Hemagglutinin expressed in transfected human cells. Glycobiology. 2018; 28: 42–49. 10.1093/glycob/cwx092 29087468

[25] Angulo J, Enriquez-Navas P.M, Nieto PM. Ligand–Receptor Binding Affinities from Saturation Transfer Difference (STD) NMR Spectroscopy: The Binding Isotherm of STD Initial Growth Rates. Chem. Eur. J. 2010; 16: 7803–7812. 10.1002/chem.200903528 20496354

[26] Vasile F, Reina JJ, Potenza D, Heggelund JE, Mackenzie A, Krengel U, et al Comprehensive analysis of blood group antigen binding to classical and El Tor cholera toxin B-pentamers by NMR. Glycobiology. 2014; 24:766–78. 10.1093/glycob/cwu040 24829308

[27] Monaco S, Tailford L E, Juge N, Angulo J. Differential Epitope Mapping by STD NMR Spectroscopy To Reveal the Nature of Protein–Ligand Contacts. Angew. Chem. Int. Ed. 2017; 56: 15289–15293. 10.1002/anie.201707682 PMC5725711 28977722

[28] Haussinger D, Ahrens T, Sass H.J, Pertz O, Engel J, Grzesiek S. Calcium-dependent Homoassociation of E-cadherin by NMR Spectroscopy: Changes in Mobility, Conformation and Mapping of Contact Regions. J. Mol. Biol. 2002; 324: 823–839. 12460580 10.1016/s0022-2836(02)01137-3

[29] Case DA, Betz RM, Cerutti DS, Cheatham TE, Darden TA, Duke RE, et al AMBER 2016, University of California, San Francisco

[30] Lindorff-Larsen K, Piana S, Palmo K, Maragakis P, Klepeis JL, Dror RO et al Improved side-chain torsion potentials for the Amber ff99SB protein force field. Proteins. 2010; 78:1950–1958. 10.1002/prot.22711 20408171 PMC2970904

[31] Epik, version 2.2, Schrödinger, LLC, New York, NY, 2011.

[32] Chang G, Guida WC, Still WC. An internal-coordinate Monte Carlo method for searching conformational space. J. Am. Chem. Soc. 1989; 111: 4379–4386.

[33] Glide version 7.0, Schrödinger, LLC, New York, NY, 2016.

[34] Doro F, Saladino G, Belvisi L, Civera M, Gervasio FL. New Insights into the Molecular Mechanism of E-Cadherin-Mediated Cell Adhesion by Free Energy Calculations. J. Chem. Theory Comput. 2015; 11: 1354−1359. 10.1021/ct5010164 26574347

[35] Jorgensen WL, Chandrasekhar J, Madura J, Impey RW, Klein ML. Comparison of simple potential functions for simulating liquid water. J. Chem. Phys. 1983; 79: 926–935.

[36] Vanquelef E, Simon S, Marquant G, Garcia E, Klimerak G, Delepine JC, R.E.D. Server: a web service for deriving RESP and ESP charges and building force field libraries for new molecules and molecular fragments. Nucl. Acids Res. 2011; 39: W511–W517. 10.1093/nar/gkr288 21609950 PMC3125739

[37] Berendsen HJC, Postma JPM, van Gunsteren WF, DiNola A, Haak JR. Molecular dynamics with coupling to an external bath. J. Chem. Phys. 1984; 81:3684–3690.

[38] Grest GS, Kremer K. Molecular dynamics simulation for polymers in the presence of a heat bath. Phys. Rev. A. 1986; 33:3628–3631; 10.1103/physreva.33.3628 9897103

[39] Sindhikara DJ, Kim S, Voter AF, Roitberg AE. Bad Seeds Sprout Perilous Dynamics: Stochastic Thermostat Induced Trajectory Synchronization in Biomolecules. J. Chem. Theory Comput. 2009; 5:1624–1631. 10.1021/ct800573m 26609854

[40] Ryckaert JP, Ciccotti G, Berendsen HJ. Numerical Integration of the Cartesian Equations of Motion of a System with Constraints: Molecular Dynamics of n-Alkanes. Comput. Phys. 1977; 23: 327–341.

[41] Neuhaus D, Williamson M.P.; The Nuclear Overhauser Effect, In Structural and Conformational Analysis. 2000; Whiley-VCH, ISBN 0-471-24675-1.

[42] Vasile F, Civera M, Belvisi L, Potenza D, Tiana G. Thermodynamically–weighted conformational ensemble of cyclic RGD peptidomimetics from NOE data. The Journal of Physical Chemistry. 2016; 120:7098–7107. 10.1021/acs.jpcb.6b04941 27387008

