# Supplementary material for: Exploring E-cadherin-peptidomimetics interaction using NMR and computational studies
Source: PLoS Comput Biol. 2019 Jun 3;15(6):e1007041. doi: 10.1371/journal.pcbi.1007041 (PMC6564044; doi:10.1371/journal.pcbi.1007041)
Supplement: S1 Text — (DOCX) [file pcbi.1007041.s001.docx]

**NMR and computational data of ligands in the free and bound states**

**NMR conformational analysis**

The NMR assignment of compound **1** and **2** is reported in the tables S1-S4. Since molecules are not rigid, we used NMR to perform the conformational analysis and to observe the presence of multiple conformations in equilibrium. To this end, we analyzed the associated NMR parameters such as chemical shift and temperature variation of the amide protons and NOEs.

The most important factor in the application of NMR spectroscopy to conformational analysis is the existence of a straightforward relationship between the nuclear Overhauser enhancement (NOE) and the distance between nuclei. NOEs (which are due to the transfer of nuclear spin polarization from one spin to another spin via cross relaxation) are used to determine the conformational preference of a molecule in solution. *Variable Temperature NMR (VT-NMR)*. In diluted solution, the chemical shift of exchangeable protons (-OH, -NH or NH_2_) depends dramatically on the extent of both the inter-molecular and the intra-molecular hydrogen bonding network. When the temperature changes, the populations of available conformations change; as a consequence, the number of intra-molecular hydrogen bonds varies, causing dramatic changes in the chemical shifts of -NH resonances. In particular, when the solvent used for the experiments is water, with the increasing of the temperature, the signals of exchangeable, solvent exposed protons shift towards high fields because of the reduction in the extent of the hydrogen bonding network. Generally, it is possible to say that a proton is engaged in an intra-molecular hydrogen bond when the ratio Δδ/ΔT is less than 5.

**Compound 1.** The complete analysis of the NOE contacts ligand **1** at 298K suggested an equilibrium between different conformations. The presence of a long range interaction between the α proton of the aspartic side chain and the tert-butyl moiety suggested the presence in solution of a closed conformation. By the analysis of the VT experiments, we can see that the proton of NH_10_ is the only one that could be engaged in an inter-molecular hydrogen bond (Table S5 and Figure S1).

The interaction between the tert-butyl moiety and the α proton of the aspartic side chain is conserved at both the lower and at the higher temperature.

**Compound 2.** The NOESY spectra did not show any significant long range NOE contact typical of a preferred conformation at both 283 K and 298 K and the exchangeable protons are not involved in intra-molecular hydrogen bond formation.

Computational conformational analysis.

Epik module of Schrodinger suite [31] was applied to predict the ligands protonation state at physiological condition (pH=7 and water solution). In these conditions, compound **1** and **2** are zwitterions with charged N-terminal amino group and a deprotonated aspartic acid.

For compound **1**, relevant conformations from Monte Carlo/Multiple Minimization (MC/MM) calculations [32] were selected for Monte Carlo/Stochastic Dynamics (MC/SD) simulations (F. Guarnieri and W. C. Still, J. Comp. Chem., 1994, 15, 1302) and the computational results were compared to the NMR data.

For compound **2**, to sample 8,5-lactam ring geometries, MC/MM were performed starting from the neutral and charged states (Fig. S7). In fact, according to Epik, the tertiary scaffold amine (predicted pKa 7.7) is likely to exist as neutral and protonated forms, equally populated. The most relevant lactam ring geometries were used as input conformations for docking runs. Among the charged forms, only the energetically more stable NH(R)+, was selected for docking calculations.

MC/MM protocol for compound 1 and 2. All calculations were performed using Macromodel version 9.9. For both compounds MC/MM conformational searches were carried out using the AMBR* (S. J. Weiner et al., J. Comput. Chem., 1986, 7, 230) and OPLS_2005(J. L. Banks et al., [J. Comp. Chem., 2005, 26, 1752](http://pubs.acs.org/cgi-bin/abstract.cgi/jmcmar/2004/47/i07/abs/jm0306430.html)) force fields and the implicit water GB/SA solvent model (W. C. Still et al., J. Am. Chem. Soc., 1990, 112, 6127). To delete side chains effects on calculations, aspartic acid (Asp) and isoleucine (Ile) residues were mutated into alanine (Ala) residues and standard capping groups N-terminal acetyl (Ac) and C-terminal methyl amide (NHMe) capping groups were used. For each search, at least 1000 starting structures for each variable torsion angle were generated and minimized until the gradient was less than 0.05 kJ Å−1 mol−1 using the truncated Newton–Raphson method (J. W. Ponder and F. M. Richards, J. Comp. Chem., 1987,8, 1016).

Only trans amide were allowed and duplicate conformations (considered different if the maximum distance between corresponding atoms following optimal rigid-body superposition is > 0.5Å) and those with an energy greater than 6 kcalmol−1 above the global minimum were discarded.

MC/SD protocol for compound 1. Relevant geometries of the MC/MM calculations were used as starting points for MC/SD simulations in implicit GB/SA water solvent. Calculations were carried out using AMBER* and OPLS_2005 force fields after a structure minimization (TNGC algorithm, 0.05 kJÅ−1mol−1 gradient convergence) of the complete molecule (with proper residues and capping group) and an equilibration step to 300K (10 ps, time step 0.5 fs). A cutoff of 20 Å was applied for the non-bonded interactions. Dihedral angles were defined as internal coordinate degrees of freedom in the Monte Carlo part of the algorithm. A time step of 1 fs was used for the stochastic dynamics (SD) part of the algorithm for 20 ns of simulation time. Samples were taken at 4 ps intervals during each simulation, yielding 5000 conformations for analysis.

**Compound 1 MC/MM and MC/SD results.** Representative conformers (i.e. structures found within 3 kcal/mol from the global minimum) obtained using AMBER* force field converged to the same folded structure characterized by a 12-membered hydrogen bond between the backbone NH and CO groups of Ala residues emerging from the opposing DKP arms. The NMR 10-membered hydrogen bond geometry was found at 4.106 kcal/mol from the global minimum and selected as starting point for MS/SD simulation. The 12-membered geometry was the only conformation sampled during the simulation (98% of the saved structures, see Figure S8 left panel).

On the contrary, MC/MM conformers obtained using OPLS_2005 force field showed a great variety of folded geometries. In the global minimum geometry, the methyl amide capping group forms a hydrogen bond with the carbonyl of DKP (see Fig S8, center panel). Interestingly, the conformation with the 10-membered ring hydrogen bond involving NH10 was found at 1.486 kcal/mol from the global minimum (Fig. S8, right panel) while the 12-membered, at 2.635 kcal/mol, resulted less stable.

The global minimum and the 10-membered ring conformations were selected as starting geometries for MC/SD simulations. Both calculations identified the hydrogen bond between DKP-C=O4 and NH-t-butyl amide of the global minimum structure as the most populated (36% and 30% of the sampled structure with a distance cutoff of 3 Å). The 10-membered ring hydrogen is less populated (0.5% and 0.3% for a distance cutoff of 3 Å that increases up to 3% and 2.5% with a threshold of 4.5 Å) indicating together with the average distance of the Asp-Hα/NH-t-butyl NOE contact (10.794±1.511 Å and 10.859±1. 481 Å) that the molecule mainly adopted an extended geometry.

**Compound 2 MC/MM results.** MC/MM conformational searches were carried out on the charged NH(R)+ and NH(S)+ and neutral states**.** NH(R)+ resulted the most stable protonated form. In fact, according to MC/MM results with OPLS_2005 force filed, the energy difference between the global minimum of NH(R)+ and NH(S)+ is 2.08 kcal/mol and with AMBER* force filed NH+(S) corresponded only to the 18% of the Boltzmann-weighted conformers at 298.15 K (was not a global minimum). As consequence, NH+(R) was selected as the charged form for docking calculations.

All the calculations converged to the same global minimum ring geometry regardless of the force field and protonation state. This ligand ring conformation was used for docking calculation in E-model. A second 8,5-lactam ring geometry was found (only with AMBER* force field), corresponding to the 21% (for NH(R)+) and 9%(for the neutral form) of the Boltzmann-weighted conformers population at 298.15 K. This less important ring conformations was also used in docking calculations of the neutral form into E-cadherin model (S9 Fig) showing similar results of the corresponding global minimum ring geometry.

**Comparison of compound 1 docking poses with STD-NMR data**. The docking poses were analyzed on the basis of STD data to select a starting geometry for MD simulations. In the best pose (Fig 4) NH_1_ formed a hydrogen bond with the backbone of Trp2 (STD signal at 283 K and 290 K), NH_10_ is close to Glu89 (STD signal at 283 K) and the NH_Ile_ proton is solvent exposed (STD signal at 290 K and 298 K). In other docking poses the NH_Ile_ proton forms a HB with the backbone of Trp2. This type of arrangement, where all the STD protons are in contacts with the protein, was selected for MD runs.

**Comparison of compound 2 docking poses with STD-NMR data**. Both docking binding modes **A** and **B** (Fig 5) are in agreement with the NMR-STD ligand epitope: they anchor the benzyl group into the Trp2 hydrophobic pocket giving evidence of the aromatic hydrogen signals (detected in all the recorded STD spectra), and place the NH_19_ (STD signals at 283 K and 290 K) and NH_2_ protons (always detected) close to the E-cadherin residues. In particular, NH_19_ proton in **A** is close to the side chain of Asn27 and Glu89 while in **B** is surrounded by Asp90, Asp1 and Pro91; NH_2_ protons in **A** are in contacts with the side chain of Asn27 while in **B** form hydrogen bonds with Glu89 side chain.

**MD simulations input preparation**

**Model preparation**. The EC1 domain structure was taken from the X-ray swap dimer complex (3q2v.pdb) and prepared as previously reported [18]. All crystallographic waters were deleted and no capping groups were added. The same receptor structure was used for docking calculations and MD simulations. For the MD simulations the two Ca2+ ions at the C-terminus of EC1 domain were modeled according to BradBrook parameters (G. M. Bradbrook et al., J. Chem. Soc., Faraday Trans., 1998, 94, 1603).

**Ligands preparations**. All the ligands were considered as zwitterion and AMBER99SBildn* force field [30] was used.

Compound 1**.** This molecule contains two non-standard parts, the DKP scaffold and the t-butyl capping group, that were prepared as new units using the R.E.D. software [36] (for the generation of RESP charges) and the antechamber module of AmberTool16 [29]. As first step two simplified model molecules were generated: the DKP scaffold capped with acetyl and methyl amide groups at the C- and N-termini and the t-butyl moiety connected to an acetyl group. Using the Ante_RED-1.5.pl script, the .pdb file of the model molecules were then converted into the corresponding Gaussian09 (M. J. Frisch et al., Gaussian, Inc., Wallingford CT, 2009). files for geometry optimization and calculation of Molecular electrostatic Potential (HF/6-31G* level). R.E.D input files (.p2n). with the INTRA-MCC option for the capping groups (no charges calculation) and two different spatial orientations of the molecules were applied (highly reproducible RESP charges independent from the initial Cartesian coordinates). The RESP charges were generated using the RED-vIII.5.pl script with the RESP-A1 option (HF/6-31G*). The output file (.mol2), containing the coordinates and charges of the new units, where manually edited to set amber99SB*ildn force filed atom types and the antechamber utility was applied to derive the amber .prep files.

Two missing angle parameters of the DKP benzyl group (namely CA-CT-H1 and CA-CT-N) were detected and the corresponding gaff force field (J. Wang et al., J. Comp. Chem., 2004, 25, 1157) parameters (ca-c3-h1 and ca-c3-n) were implemented.

Compound 2**.** The non-standard azabicycloalkane scaffold was prepared as a new unit applying the same protocol described for the DKP scaffold. Two separate sets of charges were calculated for the neutral and the positively charged of 8,5-lactam ring. The scaffolds were capped with acetyl and methyl amide groups.

Two missing angle parameters of the benzyl group were detected and the corresponding gaff force field parameters were implemented.
