# Supplementary figures and images for: Exploring E-cadherin-peptidomimetics interaction using NMR and computational studies
Source: PLoS Comput Biol. 2019 Jun 3;15(6):e1007041. doi: 10.1371/journal.pcbi.1007041 (PMC6564044; doi:10.1371/journal.pcbi.1007041)

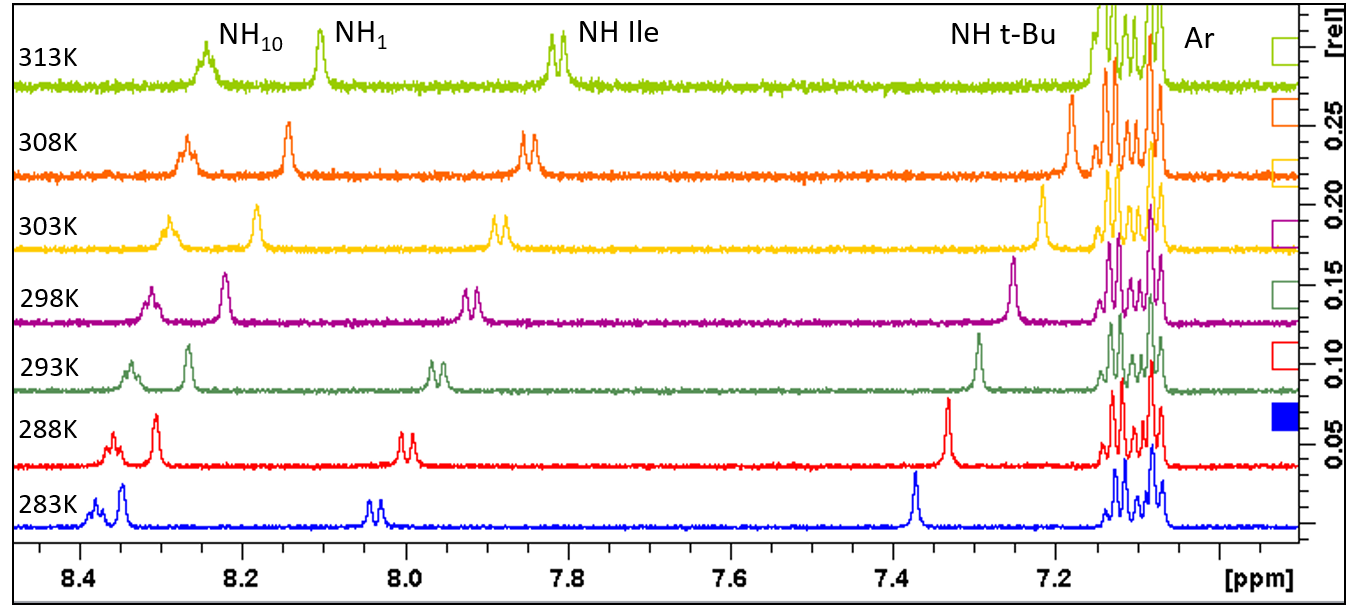

Supplement: S1 Fig — The interaction between the tert-butyl moiety and the α proton of the aspartic side chain is conserved at both the lower and at the higher temperature. (TIF) [file pcbi.1007041.s002.tif]

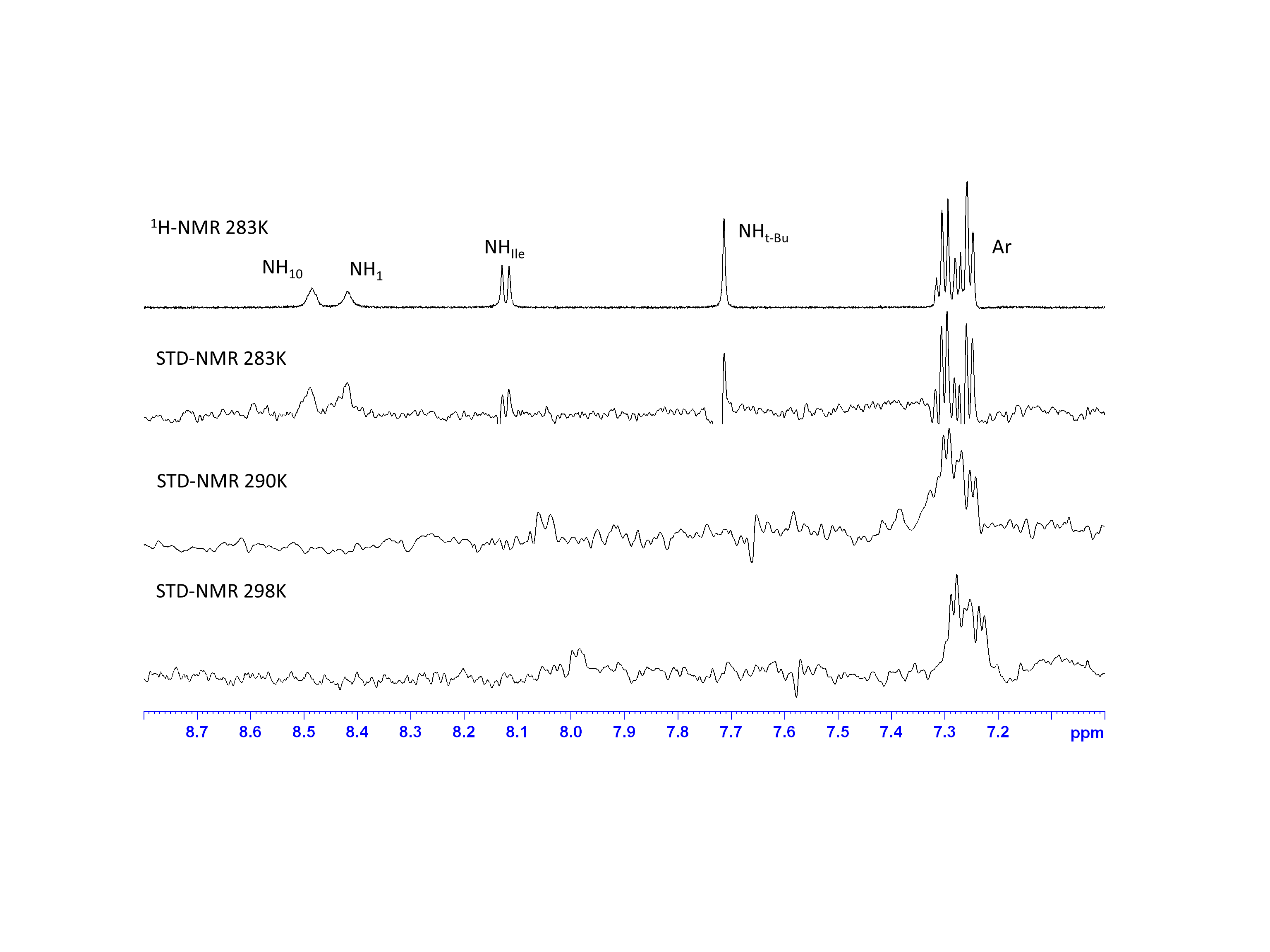

Supplement: S2 Fig — (TIF) [file pcbi.1007041.s003.tif]

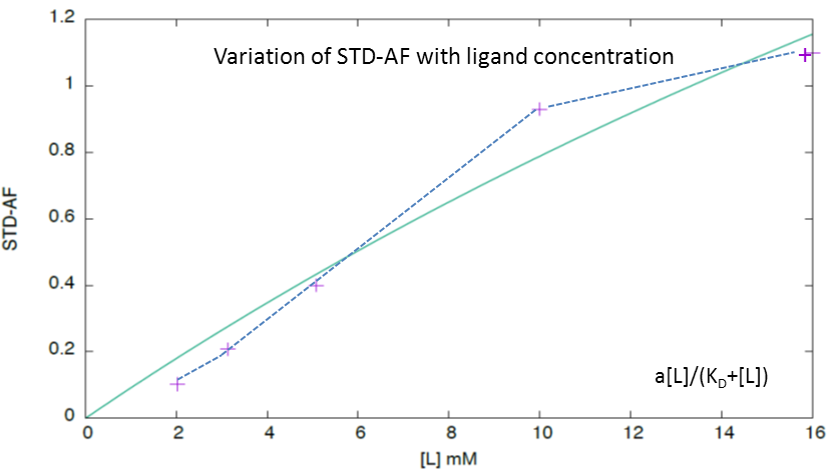

Supplement: S3 Fig — We measured the KD value of compound 1 (S3 Fig) and 2 in the presence of E-cadherin by STD (for each point we performed two STD measurements). We obtained a KD value of more than 20 mM, demonstrating a low affinity of this compound for the protein. (TIF) [file pcbi.1007041.s004.tif]

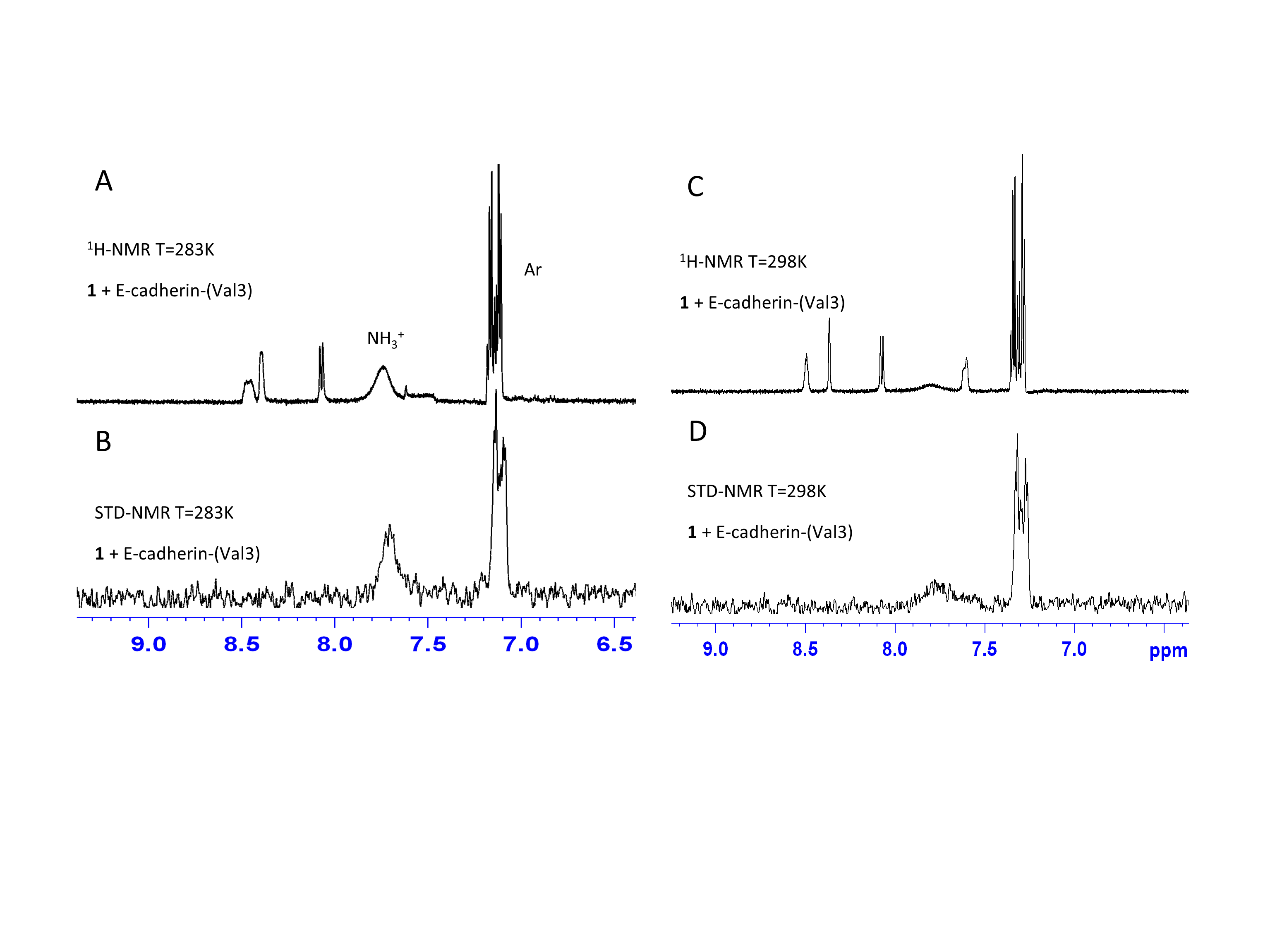

Supplement: S4 Fig — A) and C) 1H-NMR at 283 K and 298 K of compound 1 in the presence of E-cadherin-(Val3)-EC1EC2, respectively. B) and D) STD-NMR at 283 K and 298 K of compound 1 in the presence E-cadherin-(Val3)-EC1EC2, respectively. The observation of the terminal AspNH3+ is possible since we acquired experiments in the absence of D2O. (TIF) [file pcbi.1007041.s005.tif]

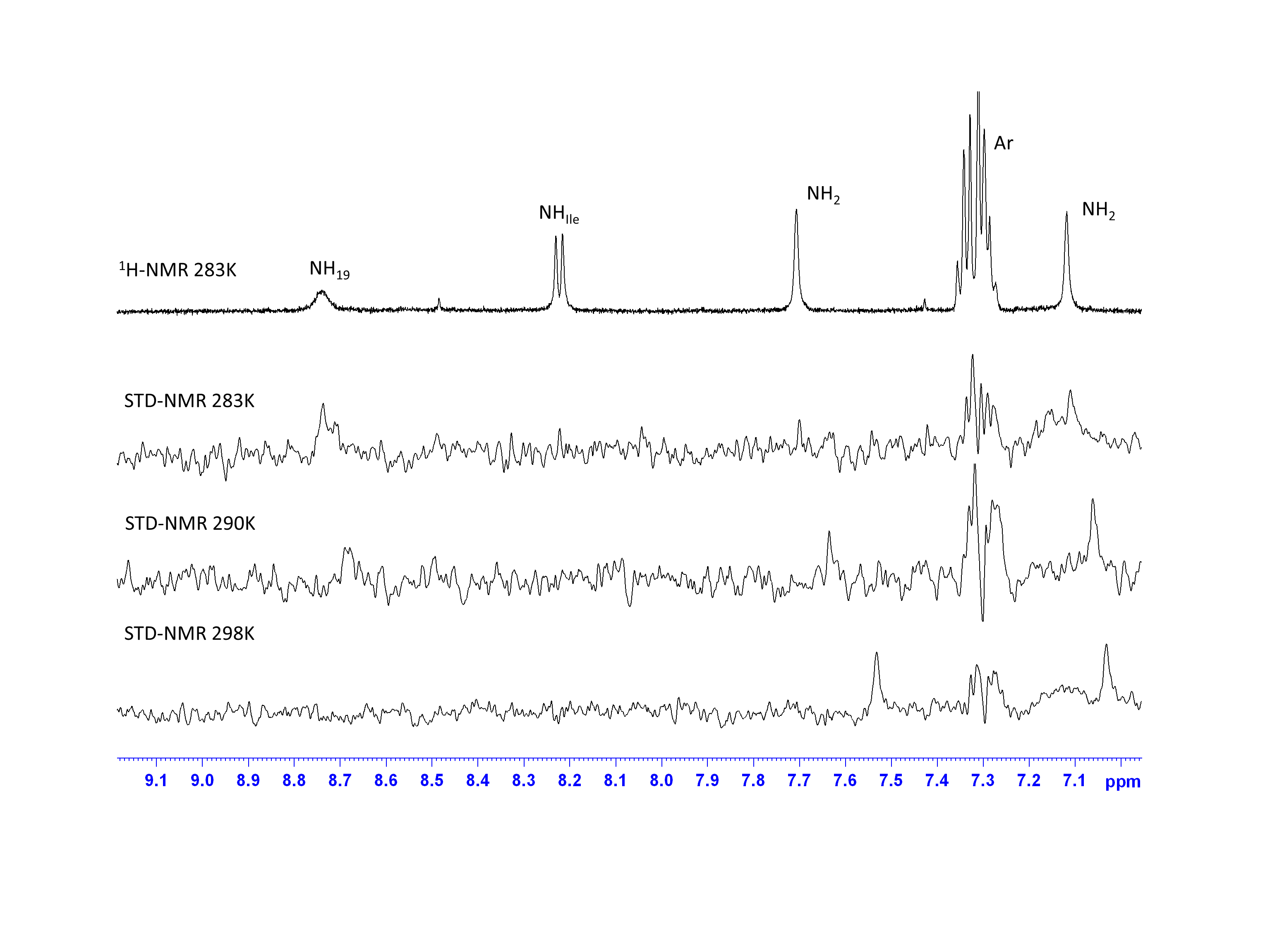

Supplement: S5 Fig — (TIF) [file pcbi.1007041.s006.tif]

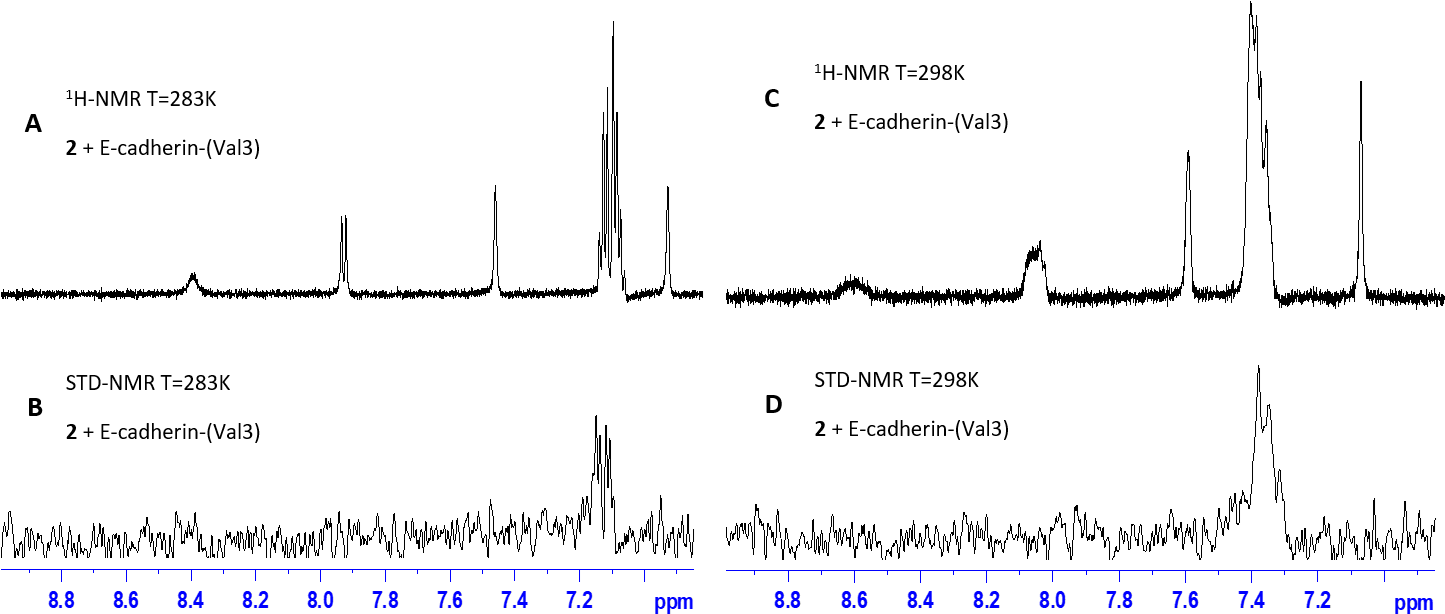

Supplement: S6 Fig — A) and C) 1H-NMR at 283 K and 298 K of compound 2 in the presence of E-cadherin-(Val3)-EC1EC2, respectively. B) and D) STD-NMR at 283 K and 298 K of compound 2 in the presence E-cadherin-(Val3)-EC1EC2, respectively. (TIF) [file pcbi.1007041.s007.tif]

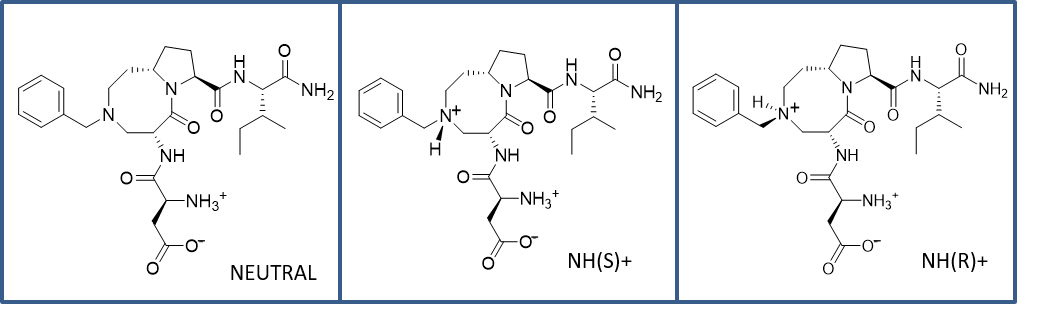

Supplement: S7 Fig — According to Epik [31], the tertiary scaffold amine of compound 2 (predicted pKa 7.7) is likely to exist as neutral and protonated forms, equally populated, at physiological condition (pH = 7 and water solution). (TIF) [file pcbi.1007041.s008.tif]

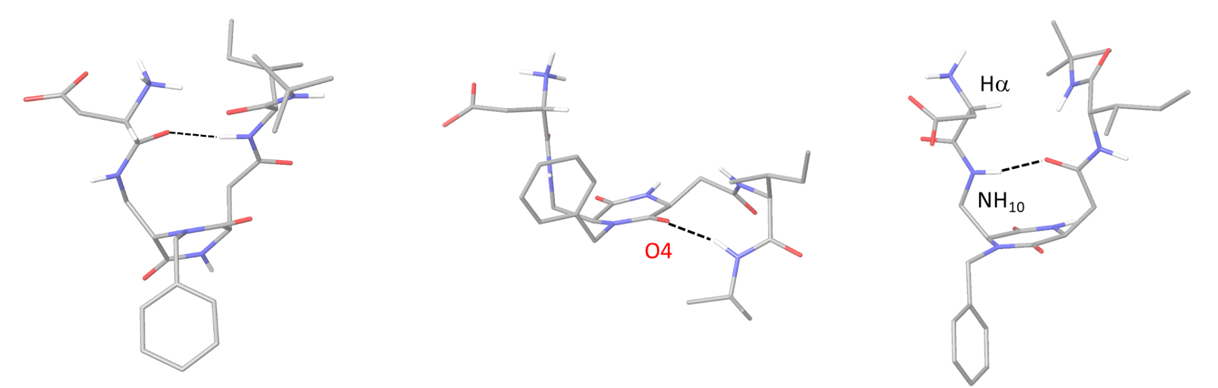

Supplement: S8 Fig — Left: Most populated 12-membered ring hydrogen bond geometry sampled with AMBER* during MC/SD simulation; Center: MC/MM OPLS_2005 global minimum geometry; Right: 10-membered ring hydrogen bond structure. (TIF) [file pcbi.1007041.s009.tif]

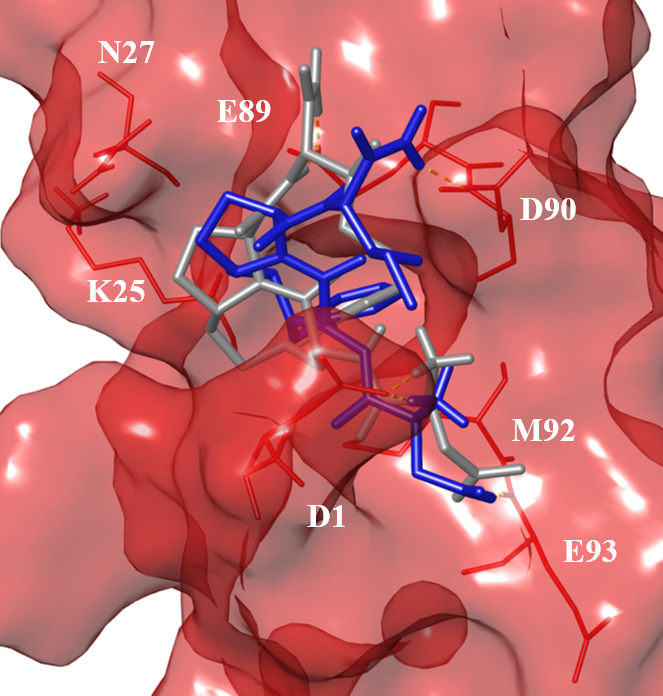

Supplement: S9 Fig — Ligand global minimum ring geometry (grey) and the relative minimum geometry (blue) were shown. (TIF) [file pcbi.1007041.s010.tif]

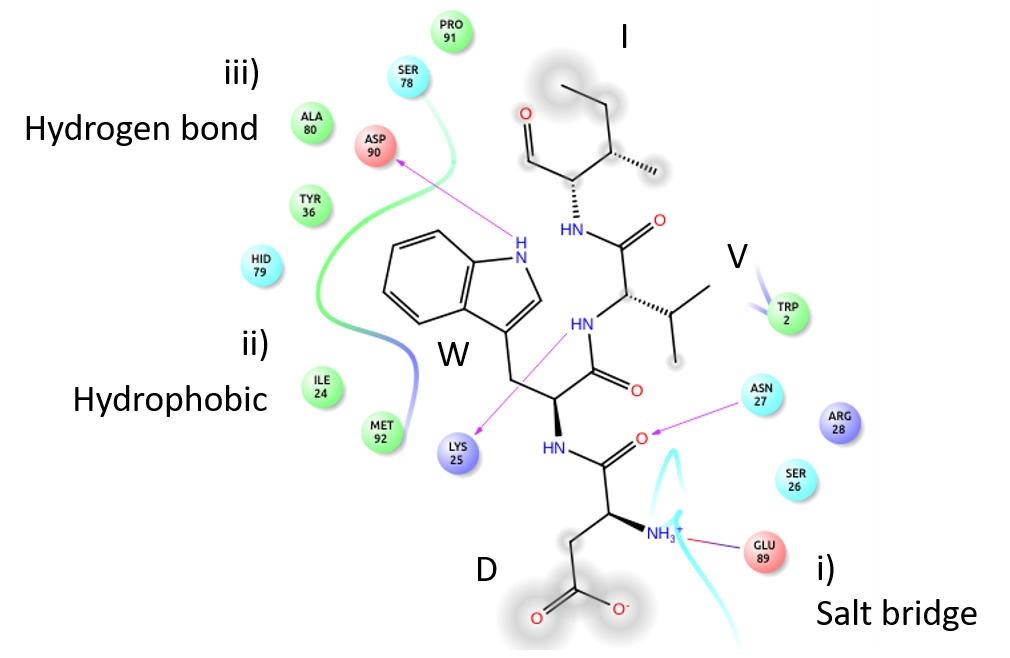

Supplement: S10 Fig — The E-cadherin interactions of the DWVI sequence in the X-ray structure of the swap dimer are formed by an intermolecular salt bridge between the charged N-terminal amino group of Asp1 and the side chain of Glu89 (i), the anchoring of the aromatic moiety of Trp2 into a hydrophobic pocket (ii) and the hydrogen bond between the indole NHε and the carbonyl group of Asp90 backbone (iii). Protein residues within 4 Å are shown, PDB CODE: 3Q2V. (TIF) [file pcbi.1007041.s011.tif]

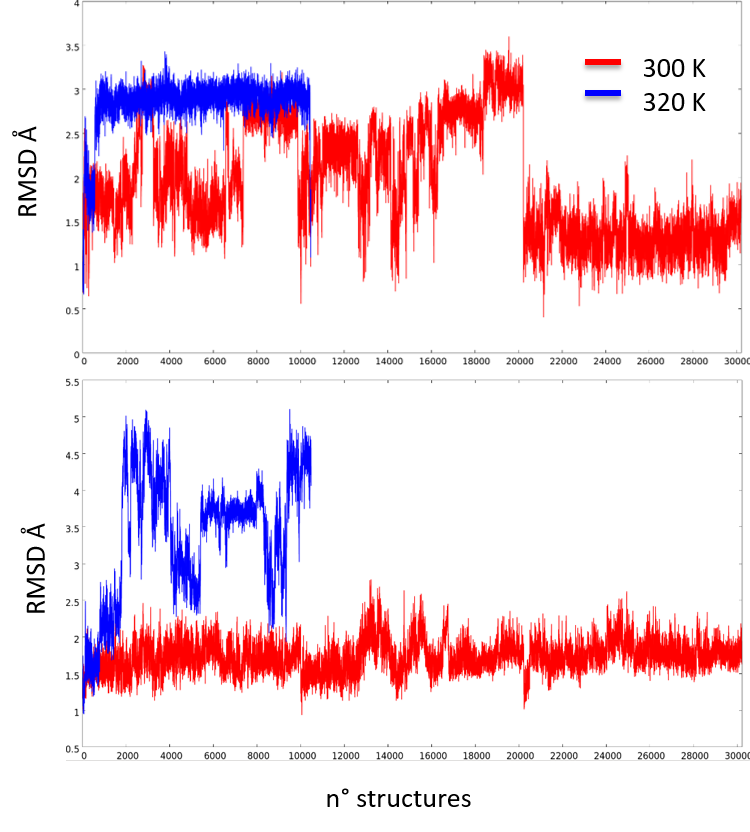

Supplement: S11 Fig — Ligand heavy atoms root-mean-square deviation (RMSD, upper level) and protein backbone atoms (C, O, N, Cα, H) RMSD (lower level) of compound 1 calculated with respect to the docking pose at 300 K (red) and 320 K (blue). (TIF) [file pcbi.1007041.s012.tif]

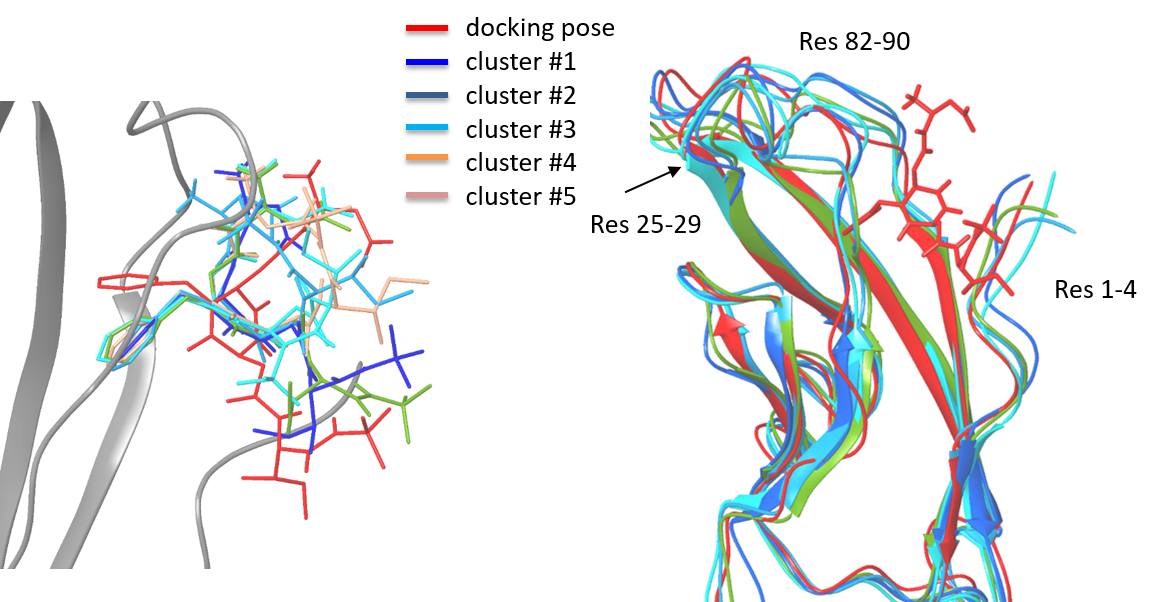

Supplement: S12 Fig — Left: ligand clusters on heavy atoms (#1 = 35%, #2 = 21%, #3 = 14%, #4 = 12% and #5 = 6%) overlaid to the starting geometry (red); Right: protein clusters on Cα atoms (#1 = 40%, #2 = 24%, #3 = 14% and #4 = 6%) overlaid to the starting geometry (red). Flexible loop and adhesive arm residues are indicated. (TIF) [file pcbi.1007041.s013.tif]

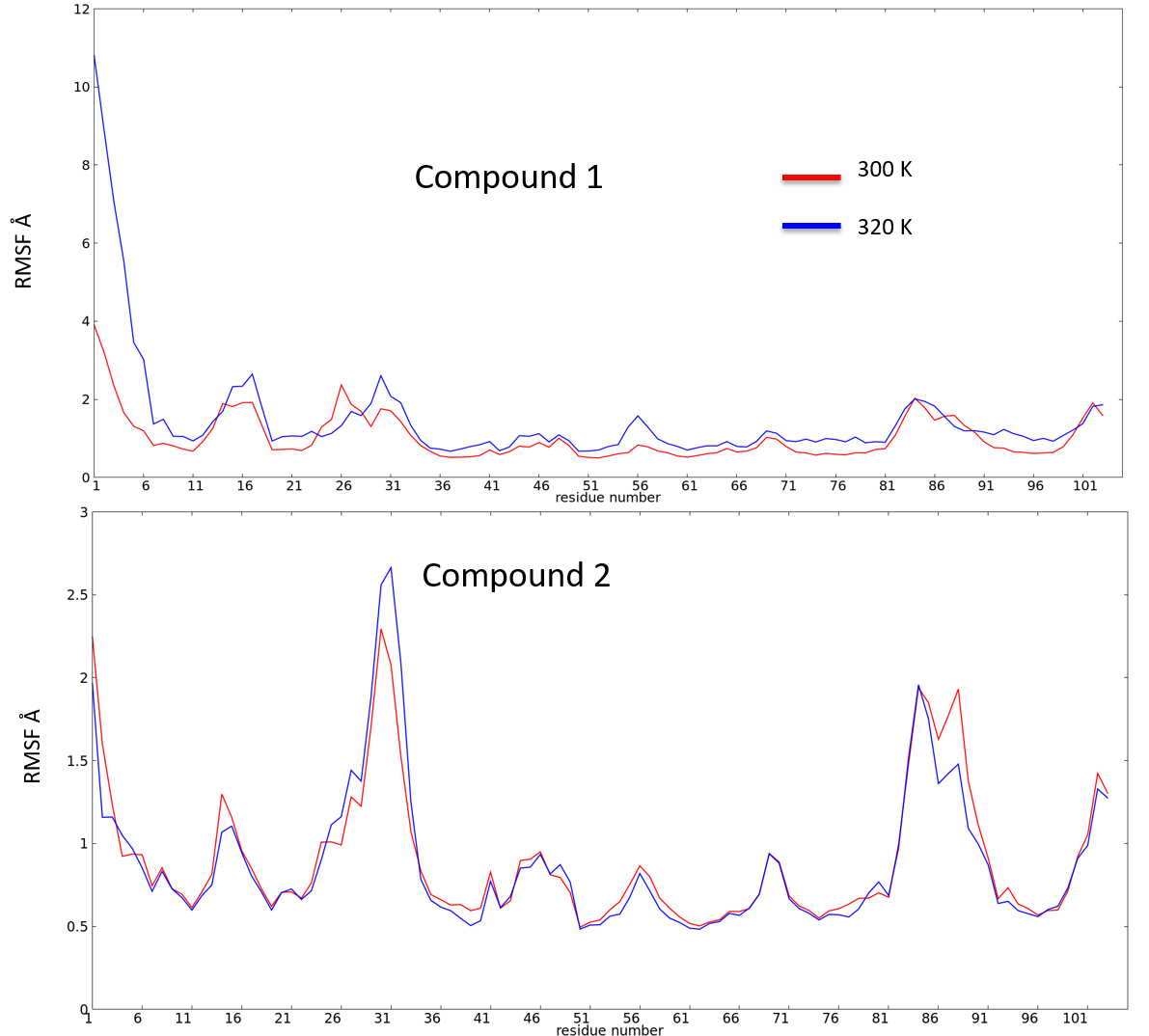

Supplement: S13 Fig — Protein root-mean-square fluctuation (RMSF C, O, N, Cα, H backbone atoms) of compounds 1 (upper panel) and 2 (lower panel) calculated with respect to the x-ray structure at 300 K (red) and 320 K (blue). (TIF) [file pcbi.1007041.s014.tif]

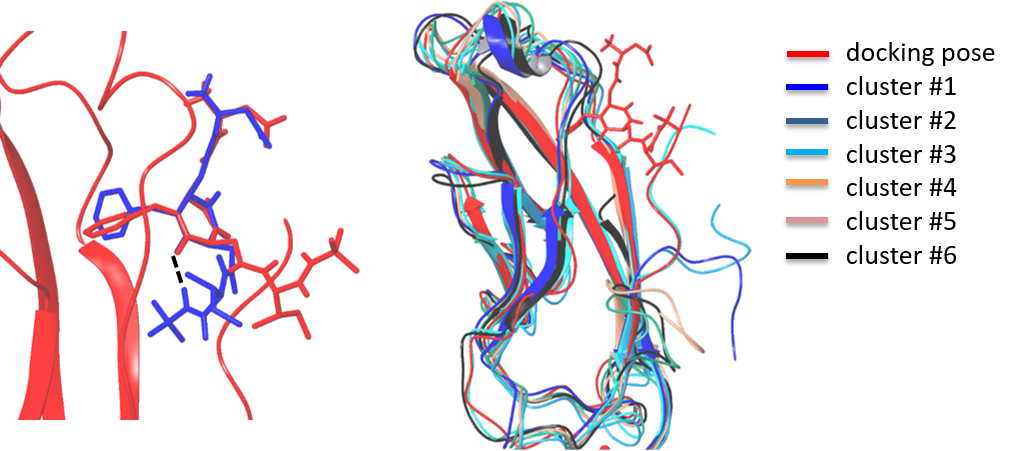

Supplement: S14 Fig — Left: most populated ligand cluster (#1 = 92%) overlaid to the starting geometry (red); Right: protein clusters on Cα atoms (#1 = 20%, #2 = 10%, #3 = 8%, #4 = 7%, #5 = 7% and #6 = 6%,) overlaid to the starting geometry (red). (TIF) [file pcbi.1007041.s015.tif]

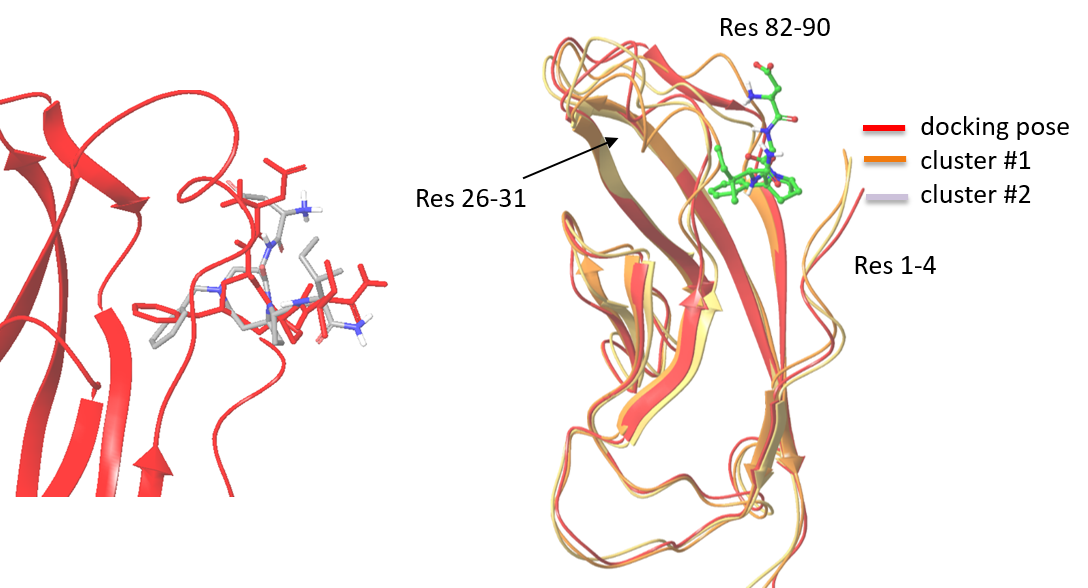

Supplement: S15 Fig — Left: ligand cluster (on heavy atoms, 99% populated) overlaid to the starting geometry (red); Right: protein clusters (on Cα atoms) overlaid to the starting geometry (red). Flexible loop and adhesive arm residues are indicated. (TIF) [file pcbi.1007041.s016.tif]

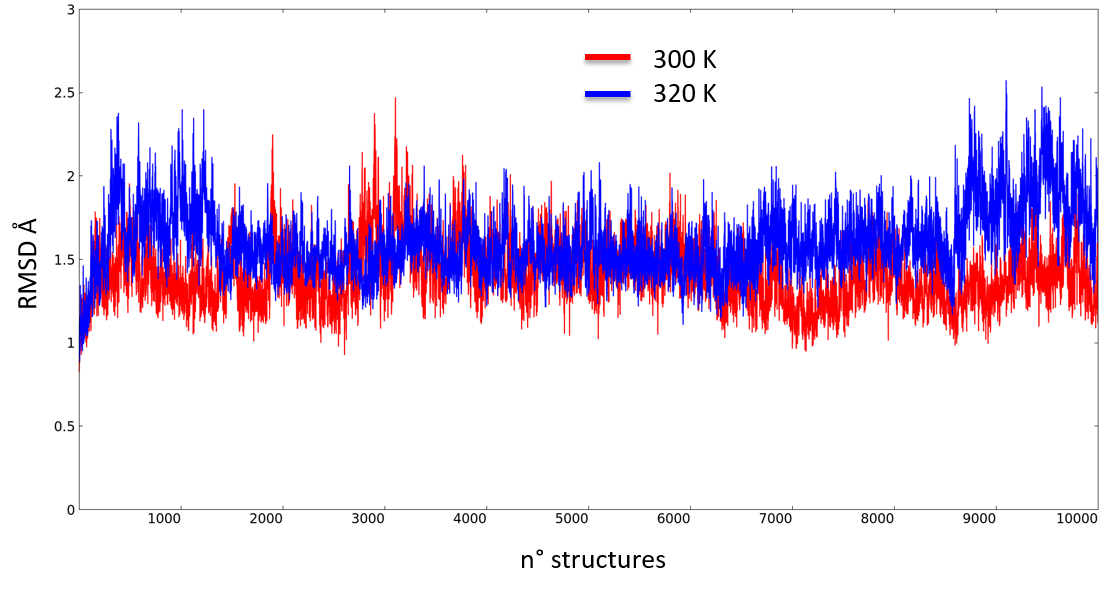

Supplement: S16 Fig — (TIF) [file pcbi.1007041.s017.tif]
