# Supplementary material for: Exploring E-cadherin-peptidomimetics interaction using NMR and computational studies
Source: PLoS Comput Biol. 2019 Jun 3;15(6):e1007041. doi: 10.1371/journal.pcbi.1007041 (PMC6564044; doi:10.1371/journal.pcbi.1007041)
Supplement: S1 Table — (PDF) [file pcbi.1007041.s018.pdf]

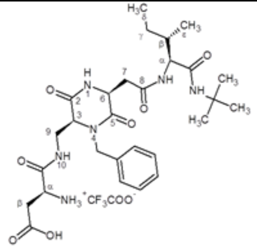	^{1}H (δ,ppm)	^{13}C (δ,ppm)	NOE free
NH_1	8.42	/	
H_3	4.29	59.00	Ar(w), H_2C-Ar(w)
H_6	4.44	51.40	
H_7	2.83/3.06	37.66	NHLeu(m)
H_9	3.52/3.74	40.90	Ar(w)
NH_{10}	8.54	/	
αAsp	4.06	51.20	NHtBu(w), tBu(w)
βAsp	2.40/2.60	37.74	
NHIle	8.11	/	H_7(m)
αIle	3.94	58.50	NHtBu(m)
βIle	1.69	36.40	
γ_1Ile	1.12/1.40	24.90	
δIle	0.79	9.66	Ar(w)
γ_2Ile	0.84	14.58	
NHtBu	7.65	/	αAsp(w), αIle(m), βIle(w), γ_2Ile(w)
tBu	1.23	27.98	
H_2C-Ar	4.63		H_3
Ar	7.30	128.40	H_9(w), γ_2Ile(w)
