# Supplementary material for: Exploring E-cadherin-peptidomimetics interaction using NMR and computational studies
Source: PLoS Comput Biol. 2019 Jun 3;15(6):e1007041. doi: 10.1371/journal.pcbi.1007041 (PMC6564044; doi:10.1371/journal.pcbi.1007041)
Supplement: S2 Table — (PDF) [file pcbi.1007041.s019.pdf]

	^{1}H (δ,ppm)	^{13}C (δ,ppm)	NOE free
NH_1	8.38	/	
H_3	4.15	58.83	Ar(w)
H_6	4.26	51.35	
H_7	2.64/2.91	37.22	NHIle(s)
H_9	3.33/3.55	40.73	
NH_{10}	8.46	/	αAsp(m)
αAsp	3.88	50.89	NH_{10}(m), NHtBu(w), tBu(w)
βAsp	2.20/2.43	37.25	
NHIle	8.05	/	H_7(m)
αIle	3.75	58.17	NHtBu(m)
βIle	1.51	35.85	-
γ_1Ile	0.95/1.23	24.94	-
δIle	0.61	9.36	
γ_2Ile	0.67	14.68	NHtBu(w)
NHtBu	7.60	/	αAsp(w), αIle(s), γ_2Ile(w)
tBu	1.05	27.75	αAsp(w)
H_2C-Ar	4.38/4.53	49.36	
Ar	7.11	127.9	βIle(w), γ_2Ile(w)
