# Supplementary material for: Exploring E-cadherin-peptidomimetics interaction using NMR and computational studies
Source: PLoS Comput Biol. 2019 Jun 3;15(6):e1007041. doi: 10.1371/journal.pcbi.1007041 (PMC6564044; doi:10.1371/journal.pcbi.1007041)
Supplement: S3 Table — (PDF) [file pcbi.1007041.s020.pdf]

| 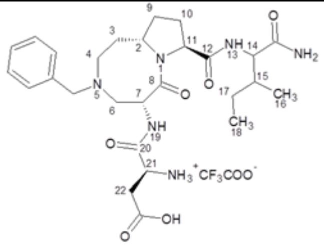 | $^{1}H$ (δ,ppm) | $^{13}C$ (δ,ppm) | NOE free |
|---|---|---|---|
| $H_2$ | 4.45 | 59.30 | |
| $H_3$ | 1.53/1.57 | 35.06 | $H_4/H_6$(w) |
| $H_4$ (*) | 2.90 | 51.30 | $H_7$(m), $H_2$C-Ar(m), Ar(w) |
| $H_6$ (*) | 2.82/2.93 | 57.35 | $H_3$(m), $H_{10}$(w), $H_2$C-Ar(m), Ar(w) |
| $H_7$ | 5.05 | 50.73 | $H_4/H_6$(m) |
| $H_9$ | 1.69/2.13 | 30.86 | |
| $H_{10}$ | 1.90/2.34 | 26.86 | |
| $H_{11}$ | 4.48 | 60.10 | |
| $NH_{13}$ | 7.97 | / | $H_9$(w), $H_{10}$(m) |
| $H_{14}$ | 4.04 | 58.00 | |
| $H_{15}$ | 1.83 | 34.70 | |
| $H_{16}$ | 0.90 | 14.60 | |
| $H_{17}$ | 1.18/1.48 | 24.30 | |
| $H_{18}$ | 0.85 | 9.50 | |
| $NH_{19}$ | 8.51 | / | |
| $H_{21}$ | 4.18 | 50.80 | |
| $H_{22}$ | 2.66/2.72 | 36.90 | |
| $H_2$C-Ar | 3.70/3.79 | 61.10 | $H_6/H_4$(s) |
| Ar | 7.35 | 129.1 | $H_3$(w) ,$H_4/H_6$(s) |
| $H_2N$ | 7.05/7.55 | / | |
