# Supplementary material for: Exploring E-cadherin-peptidomimetics interaction using NMR and computational studies
Source: PLoS Comput Biol. 2019 Jun 3;15(6):e1007041. doi: 10.1371/journal.pcbi.1007041 (PMC6564044; doi:10.1371/journal.pcbi.1007041)
Supplement: S4 Table — (PDF) [file pcbi.1007041.s021.pdf]

	^{1}H (δ,ppm)	^{13}C (δ,ppm)	NOE free
H_2	4.28	59.28	
H_3	1.39/1.33	35.11	$H_4/_6$(m)
H_4 (*)	2.72	51.61	Ar(m)
H_6 (*)	2.64/2.73	57.29	H_2(m), Ar(m)
H_7	4.89	50.63	Not visible
H_9	1.48/1.92	30.73	
H_{10}	1.70/2.14	26.91	
H_{11}	4.31	60.10	NH_{13}(m)
NH_{13}	7.95	/	H_{11}(s)
H_{14}	3.81	58.12	
H_{15}	1.62	35.11	
H_{16}	0.66	14.71	
H_{17}	0.98/1.27	24.58	
H_{18}	0.64	9.78	
NH_{19}	8.42	/	
H_{21}	3.97	50.63	
H_{22}	2.46/2.52	36.83	
H_2C-Ar	3.62/3.52	61.09	H_4/H_6(vw)
Ar	7.14	128.9	H_4/H_6(m)
H_2N	6.96/7.49	/	H_{11}(m)
