# Supplementary material for: Exploring E-cadherin-peptidomimetics interaction using NMR and computational studies
Source: PLoS Comput Biol. 2019 Jun 3;15(6):e1007041. doi: 10.1371/journal.pcbi.1007041 (PMC6564044; doi:10.1371/journal.pcbi.1007041)
Supplement: S5 Table — (PDF) [file pcbi.1007041.s022.pdf]

	$\Delta\delta/\Delta T$ (ppb*10^3)
NH_{10}	4.67
NH_1	8.33
NH_{Ile}	7.67
NH_{tBu}	7.33
