# Supplementary material for: Exploring E-cadherin-peptidomimetics interaction using NMR and computational studies
Source: PLoS Comput Biol. 2019 Jun 3;15(6):e1007041. doi: 10.1371/journal.pcbi.1007041 (PMC6564044; doi:10.1371/journal.pcbi.1007041)
Supplement: S6 Table — (PDF) [file pcbi.1007041.s023.pdf]

	283 K		290 K		298 K	
	^{1}H (δ,ppm)	Abs STD %	^{1}H (δ,ppm)	Abs STD %	^{1}H (δ,ppm)	Abs STD %
NH_1	8.38	3.623	8.61	2.137	Not visible	
NH_{10}	8.46	2.981	8.71		Not visible	
NHLeu	8.05		8.29	0.329	8.11	0.525
Ar	7.11	0.220	7.41	0.214	7.30	0.243
