# Supplementary material for: Exploring E-cadherin-peptidomimetics interaction using NMR and computational studies
Source: PLoS Comput Biol. 2019 Jun 3;15(6):e1007041. doi: 10.1371/journal.pcbi.1007041 (PMC6564044; doi:10.1371/journal.pcbi.1007041)
Supplement: S7 Table — (PDF) [file pcbi.1007041.s024.pdf]

	283 K		290 K		298 K	
	^{1}H (δ,ppm)	Abs STD %	^{1}H (δ,ppm)	Abs STD %	^{1}H (δ,ppm)	Abs STD %
$H_{4/6}$	2.72	0.265	2.90		2.90	
NH_{19}	8.42	2.078	8.51	1.441	8.51	
Ar	7.14	0.169	7.35	0.281	7.35	0.151
H_2N	6.96/7.49	0.222	7.05/7.97	0.463	7.05/7.97	0.806
