# Supplementary material for: Exploring E-cadherin-peptidomimetics interaction using NMR and computational studies
Source: PLoS Comput Biol. 2019 Jun 3;15(6):e1007041. doi: 10.1371/journal.pcbi.1007041 (PMC6564044; doi:10.1371/journal.pcbi.1007041)
Supplement: S9 Table — (PDF) [file pcbi.1007041.s026.pdf]

	**NH_{10}**	**NH_1**	**NH_{Ile}**
**Asp1(C=O)**	20%	7%	<0.5%
**Trp2(C=O)**	<0.5%	11%	8%
**Val3(C=O)**	0	<0.5%	0
