# Supplementary material for: Exploring E-cadherin-peptidomimetics interaction using NMR and computational studies
Source: PLoS Comput Biol. 2019 Jun 3;15(6):e1007041. doi: 10.1371/journal.pcbi.1007041 (PMC6564044; doi:10.1371/journal.pcbi.1007041)
Supplement: S10 Table — The starting salt bridge is highlighted in bold. (PDF) [file pcbi.1007041.s027.pdf]

**Salt bridges (ligand/protein)**	**Population (%)**	
	**300 K**	**320 K**
**Asp-NH_3+/Glu89-COO-**	4	<2%
Asp-NH_3+/Asp1-COO-	54	0
Asp-COO-/Arg28-Guan	23	72%
Asp-COO-/Asp1-NH_3+	12	0
